# The community drug distributor passport: a case study of training and recognition in Chad

**DOI:** 10.3389/fpubh.2026.1879806

**Published:** 2026-09-03

**Authors:** Sohaibou Diane, Adoum Mahamat Oumar, Barka Kali, Danny Harvey, Nurudeen Dauda, Geordie Woods, Oladele Akogun, Philip Downs

**Affiliations:** 1 Sightsavers, Haywards Heath, United Kingdom; 2 Ministère de la Santé Publique et de la Prevention, N'Djamena, Chad; 3 Organisation pour la Prévention de la Cécité, N'Djamena, Chad; 4 Modibbo Adama University of Technology, Yola, Nigeria

**Keywords:** Chad, community drug distributors, community health workers, health policy, neglected tropical diseases, universal health coverage

## Abstract

**Introduction:**

Community health workers (CHWs), particularly volunteer community drug distributors (CDDs), play essential roles in advancing universal health coverage (UHC) and neglected tropical disease (NTD) control, yet their responsibilities and formal recognition remain poorly defined across health systems. This creates policy-implementation disconnects, especially during mass drug administration (MDA) campaigns for schistosomiasis and soil-transmitted helminthiases, where CDDs deliver critical services amid declining motivation and weak community awareness.

**Methods:**

From 15 September 2023 to 1 February 2024, Sightsavers, the organization for the prevention of blindness, and Chad’s Ministry of Health and public prevention co-developed and piloted the CDD passport—a training and reference tool to clarify roles, build capacity, and formally recognize CDDs. Implemented during national MDA campaigns, the passport provided accessible materials on responsibilities, treatment protocols, and safety, alongside certificates acknowledging contributions.

**Results:**

Pre-passport assessments revealed CDDs’ limited understanding of their roles, poor community knowledge of MDA objectives, and motivational challenges. Qualitative evaluations post-pilot—with health workers, CDDs, and community members—demonstrated improved knowledge retention, enhanced perceptions of CDD legitimacy, and stronger campaign performance. Government involvement highlighted broader CHW policy gaps, including how community settlement patterns affect delivery logistics.

**Discussion:**

This community case study demonstrates how the CDD passport bridges CHW policy gaps by standardizing training, boosting motivation through recognition, and prompting national discussions on integrating volunteers into health systems. This innovation strengthens MDA effectiveness, supports sustainable NTD control, and models scalable universal health coverage (UHC) approaches for community health platforms in resource-constrained settings. Longer-term evaluation is needed to assess impacts on treatment coverage, volunteer retention, health outcomes, and cost-effectiveness.

## 1 Introduction

The term community health worker (CHW) encompasses diverse roles across countries, including volunteer workers selected and accountable to their communities—supervised by professional frontline health personnel, but not always formally part of the health system. There is ambiguity in defining their role—the International Labour Organization defines CHWs based on their activities but omits selection, supervision, and accountability dimensions (1). The World Health Organization (WHO) defines CHWs as community-based health workers with limited training, either paid or as volunteers, but also omits the context for their selection and supervision (2).

Within neglected tropical disease (NTD) programs in sub-Saharan Africa, community-directed distributors, or community drug distributors (CDDs), often fall under the CHW umbrella but are primarily focused on preventive chemotherapy through mass drug administration (MDA) campaigns and the identification of disease morbidity. WHO officially recommends regular, large-scale distribution of drugs for control and elimination of five preventive chemotherapy NTDs—lymphatic filariasis, onchocerciasis, trachoma, schistosomiasis, and soil-transmitted helminthiases. Since the late 1980s, large-scale NTD drug donation programs created the need for community-based distribution strategies, as routine health systems lacked the capacity to systematically reach all communities. During 1995–2015, the African Programme for Onchocerciasis Control (APOC) pioneered community-directed treatment with ivermectin, with non-governmental organizations (NGOs), like Sightsavers, playing a central role in assisting national programs in operationalizing the approach (3).

Originally, CDDs were selected by communities as unpaid volunteers responsible for MDA drug distribution, spending only a few weeks annually on these tasks (4). The success of the APOC community-directed treatment with ivermectin (CDTI) approach subsequently led to the expansion of CDDs’ roles to include year-round services, such as health education, community mobilization, referral, and morbidity management, aligning them more closely with generalist CHWs (5–7). Thus, the CDD role has evolved into a highly valuable community resource for health service delivery.

Despite estimates that there are 4.8 million CDDs across sub-Saharan Africa, their contributions beyond MDA remain underappreciated in global CHW policy frameworks, limiting opportunities for recognition and support. Previous studies have emphasized that policies often provide insufficient guidance on CDD roles, training, supervision, and ongoing support (8). While calls for greater recognition of CDDs within health systems have been made (4), and tools have been developed to strengthen community engagement, planning and delivery of MDA (9), few practical mechanisms have been described to operationalize such recognition within programme implementation.

Similar efforts to strengthen MDA implementation have emphasized experiential capacity-building approaches. For example, a recent trachoma programme in Afghanistan found that field-based exposure to routine delivery tools, reporting systems and supervision processes strengthened implementation capacity among health workers in a newly established programme setting (10).

This paper therefore presents a community case study focused on the process of co-creating a community volunteer training register (“CDD passport”) in Chad as an internal program innovation, implemented by the Ministry of Health in partnership with Sightsavers. The development and roll-out of a CDD Passport aimed to support CDD recognition, training and role clarification within NTD programmes.

## 2 Case context and rationale for the CDD passport

Community health worker effectiveness depends heavily on the support and formal recognition they receive from health systems. Volunteer CHWs face additional challenges in motivation and performance due to limited acknowledgment. Within NTD programs, the finite, periodic nature of MDA campaigns often diminishes the perceived value of CDDs, with emphasis on treatment delivery overshadowing other community health tasks. In the early days of the APOC programme, CDDs received in-kind or cash incentives from the community in appreciation of their services; however, this practice has diminished over time and further exacerbated demotivation and lack of recognition of this invaluable health workforce (11).

In APOC-style NTD programmes, community drug distributors are usually community-selected volunteers drawn from literate and respected local residents, including teachers, students, traders, health volunteers and committee members. In Chad, financial incentives remain a key motivator for becoming a CDD; for example, in the national program for the elimination of onchocerciasis, lymphatic filariasis, schistosomiasis, and soil-transmitted helminthiases, CDDs can receive about 2,500 CFA francs (equivalent to ~$4.50) per day provided by the national program.

Previous field observations in Chad revealed that many CDDs lacked clarity on their roles and responsibilities, and expressed frustration over limited training and recognition, factors contributing to declining motivation and inconsistent coverage. In response, the CDD passport was collaboratively developed by Sightsavers, OPC and the Ministry of Health in Chad through the national program for the elimination of onchocerciasis, lymphatic filariasis, schistosomiasis, and soil-transmitted helminthiases to support greater recognition of CDDs, including documentation of their training and description of their roles and responsibilities.

## 3 Methods

### 3.1 Case study approach and co-creation process

The CDD passport initiative was designed as a quality improvement and program-strengthening activity with an embedded evaluation and change assessment, intended solely to improve internal program functioning and not to generate generalizable scientific knowledge. The goal was to improve the knowledge, motivation, and formal recognition of volunteer CDDs in Chad engaged in MDA, initially as part of schistosomiasis and soil-transmitted helminthiases campaigns.

The process of co-creating the CDD passport was done alongside the development of educational materials to support community dialogues addressing misconceptions and harmful beliefs about NTDs and free medicines. Developed in collaboration with the Ministry of Health and local stakeholders, the CDD passport was initially produced in French and later adapted into Chadian Arabic to improve usability among CDDs. The tool was integrated into ongoing mass drug administration campaigns for schistosomiasis and soil-transmitted helminthiases. These campaigns were delivered through both school- and community-based platforms using teachers and community volunteers as CDDs.

As part of the co-creation process, qualitative data were collected in the capital towns of Amtiman and Haraze, within Salamat Province, Chad. This included key informant interviews with CDDs, district health officers, teachers, Ministry of Health representatives, non-governmental partners, community leaders (chiefs and nomadic leaders), and parents (in churches, schools, and informal settings), as well as observations during training sessions. In addition, information was supplemented by a review of national project reports and field activity observations. These methods helped identify perspectives on CDD roles, motivation, and the practical utility of a structured training and recognition tool.

In addition to qualitative methods, the evaluation incorporated quantitative data from a post-MDA Coverage Evaluation Survey (CES) to assess campaign effectiveness. The CES was conducted in 1,669 households using a structured questionnaire that captured exposure to community dialogue sessions, comprehension of key health messages, and perceived influence on treatment uptake and schistosomiasis-related behaviours among children. Data were analysed descriptively to generate indicative measures of reach, message comprehension, and perceived behavioural influence. These findings were not intended to establish causal attribution but to complement and triangulate qualitative insights from field observations, interviews, and participatory analyses. Table 1 summarizes the analytic domains and linked quantitative indicators used in the embedded evaluation.

**Table 1:** Analytic domains and corresponding Coverage Evaluation Survey (CES) indicators.

Domain	Analytic focus (qualitative)	Corresponding CES indicators (quantitative)
Barriers to volunteer engagement	Constraints affecting participation and performance of volunteer CDDs and teachers, including workload, time pressures, logistical barriers, community support, and mistrust.	No direct quantitative indicator; engagement inferred from household‑level exposure to community dialogue sessions and reported treatment acceptance.
Training quality and consistency	Quality, structure, and consistency of training for CDDs and teachers, including clarity of content, use of local languages, and application of job aids within cascade training systems.	Proportion of respondents reporting comprehension of key health messages on schistosomiasis and soil‑transmitted helminthiases.
Motivation and retention	Factors influencing CDD and teacher motivation and retention, including role clarity, perceived value of work, feedback mechanisms, and the contribution of the passport to professional identity and sustained engagement.	No direct quantitative measure; programme‑level engagement described through participation of approximately 10,000 CDDs across 54 districts.
Recognition and legitimacy	Perceptions of formal and informal recognition of CDDs (e.g., use of the passport, photographs, stamps) and views of community members, trainers, and managers regarding the legitimacy and status of CDDs as community health workers.	Not captured in the CES; assessed qualitatively through reports of high demand for the passport and descriptions of its perceived utility and completeness.
Policy and system‑level considerations	Reflections by Ministry of Health officials, provincial and departmental authorities, and implementing partners on policies governing CHWs/CDDs, and on the alignment, scalability, and sustainability of the passport within national strategies.	Not measured quantitatively; explored via key informant interviews, document review, and multi‑stakeholder design workshops.
Implementation practice and protocol adherence	Field practices during school‑ and community‑based MDA, including adherence to treatment protocols, utilization of job aids, clarity of message delivery, and management of operational delays and resource constraints.	Descriptive indicators of campaign functioning, including household reports of exposure to community dialogue sessions and perceived influence of these sessions on treatment acceptance and behaviours.
Community dialogue and communication	Quality and reach of community dialogue activities, including clarity, cultural and linguistic appropriateness of messages, and feedback from parents and caregivers on relevance and influence.	Proportion of households reporting exposure to community dialogue sessions; proportion reporting understanding of messages delivered.
Behavioural outcomes	Reported changes in behaviours relevant to schistosomiasis and soil‑transmitted helminthiases (e.g., hygiene practices, water contact, treatment‑related behaviours) and identification of persistent gaps.	Proportion of respondents indicating that community dialogue sessions influenced treatment acceptance and contributed to changes in risky behaviours
Programme performance and frontline functioning	Overall effects of the passport and associated tools on programme delivery, including role clarity, confidence among CDDs, consistency of training, community trust, and integration of volunteer CHWs within health system structures.	Combined descriptive CES indicators of message exposure, comprehension, and perceived behavioural influence, alongside programme data on CDD participation.

### 3.2 Implementation of the CDD passport

A piloted version of the passport was first explored specifically in Aboudeia and Haraze-Mangueigne Departments of Amtiman. These locations were selected because MDA activities for schistosomiasis had not yet begun, and previous MDA campaigns for trachoma had experienced low acceptance rates due to limited awareness raising amongst targeted communities. Direct field observations were made during training sessions of health center managers and CDDs on the tool. Complementary quantitative data (medication acceptance, behaviour change) and qualitative data (perceptions of hygiene, campaign comprehension, and MDA effectiveness) were obtained using a structured questionnaire deployed on the mobile-based platform CommCare. Each submission was linked to GPS coordinates, time stamps, and metadata, including health center, village, and respondent type, to support real-time, geo-referenced, and standardized analysis.

To support expansion beyond Salamat Province, the refined passport was later introduced in the capital town of Kélo and surrounding communities in Tandjilé Province, chosen due to historically low treatment coverage identified by coverage evaluation surveys. OPC staff and health centre managers administered the questionnaire, engaging in informal interviews and discussions with CDDs to capture their perspectives and experiences with the passport. Additionally, a virtual experience-sharing session was held with participation from the national NTD programme, OPC, and Kélo health centre managers, allowing for collective reflection on the passport’s utility, challenges, and observed outcomes. In total, more than 10,000 CDDs across these locations in Chad received training and a copy of the CDD passport. The French version of the CDD passport piloted in Chad during MDA campaigns for schistosomiasis and soil-transmitted helminthiases can be viewed within the Supplementary Material.

### 3.3 Analysis

Analysis was structured around the key thematic domains, including: (i) barriers to volunteer engagement; (ii) training quality and consistency; (iii) motivation and retention factors; (iv) perceptions of recognition and legitimacy; and (v) policy and system-level considerations. Field observations were guided by specific indicators, including clarity of message delivery, adherence to protocols, use of job aids (including the passport), and interaction with community members. Findings were triangulated with descriptive results from the Coverage Evaluation Survey (CES). These thematic categories were then iteratively validated through multi-stakeholder design workshops with programme staff, ministry representatives, and partners. The evaluation informed iterative refinement of the passport and contributed to policy discussions on sustainable integration of volunteer CHWs into national health systems.

Local research associates and programme staff were engaged throughout data collection and analysis to ensure contextual relevance and strengthen local monitoring and evaluation capacity. Their involvement included joint observation of trainings and drug distribution, co-facilitation of qualitative methods (interviews, empathy mapping, role-play), and collaborative review of findings. They also contributed to thematic analysis, including identification and prioritization of key barriers and enabling factors influencing volunteer engagement. This participatory approach enhanced the validity and contextual grounding of the analysis while supporting skills transfer and institutional learning for future programme adaptation and evaluation.

## 4 Learning from implementation

Approximately 10,000 CDDs participated in the CDD passport initiative across 54 districts in seven provinces in Chad. Baseline observations pointed to systemic gaps, including limited CDD understanding of NTDs, fragmented and inconsistently delivered training, and low community awareness of MDA objectives.

Following implementation, these constraints were substantially mitigated. Qualitative evidence indicates improved role clarity, more structured and applied training practices, and increased confidence among CDDs during drug distribution. These shifts are corroborated by CES data from 1,669 households, which demonstrate high levels of message exposure (89%), comprehension (over 90%), and reported influence on treatment acceptance and child behaviour. While the absence of directly comparable baseline quantitative indicators precludes causal attribution, the convergence of qualitative and quantitative evidence suggests a meaningful improvement in programme delivery, community engagement, and frontline performance associated with the passport intervention.

The passport, along with the other participatory community tools used, demonstrated high levels of community engagement, defined in this study as exposure to community dialogue sessions, understanding of key messages, treatment acceptance, and reported behaviour change. These dimensions were supported by CES findings, which showed high levels of message exposure, comprehension, and perceived influence on behaviour.

The suite of tools, including the passport, formed a community dialogue approach to bolster acceptance and efficacy of the MDA. Informants, who were primarily parents and caregivers, provided feedback on message clarity, the influence of community dialogues on drug acceptance and behaviour, and observed behavioural changes among children that reduced their risk of schistosomiasis and soil-transmitted helminthiasis.

CES data from 1,669 households indicated that 89% of respondents reported receiving information through the community dialogue sessions, and 93% confirmed understanding the messages delivered. In addition, 94% stated that community dialogue sessions influenced their acceptance of the medication, while the same proportion (based on 1,384 responses) reported that these sessions contributed to changing risky behaviours among children. These indicative signals help understand the passport’s utility.

### 4.1 Community feedback

During the initial scoping visit, field observations identified several important behavioural factors: restricted water availability and hygiene practices, the habit of taking drugs without eating beforehand, and limited understanding among participants of the activity’s purpose. Qualitative feedback from communities highlighted increased awareness of hygiene practices, with respondents frequently citing handwashing and avoiding contaminated water. Many households reported observable behaviour changes among children, while others emphasized the continued need for intensified community sensitisation and improved contextual adaptation of health messages. These findings informed revisions to the passport prior to roll-out in Kélo, including the use of simplified language, refined illustrations, and clearer sequencing of messages.

Field observations revealed persistent challenges. In several areas, hygiene infrastructure was absent (e.g., no handwashing stations), some children ingested medication without food or prior information, and CDDs were often required to improvise due to delays. Health communication relied heavily on radio broadcasts and informal community networks. These insights reinforced the importance of tailoring materials to local contexts, prioritizing local language use, and addressing structural barriers such as food insecurity and mistrust.

### 4.2 Feedback from CDDs, trainers, and partners

The passport was consistently reported to strengthen motivation among CDDs (community volunteers and teachers) by clarifying roles and responsibilities, elevating the quality of training, and fostering sustained engagement in NTD control activities. Feedback from trainers, managers, and frontline actors indicated that they highly valued the tool for its clarity, structure, and practical utility. It was the sole instructional resource used by health centre managers to train drug distributors, both teachers and non-teachers. Trainers described it as a “complete document for training”, while one CDD referred to it as a “key tool to control the distribution”. Similarly, the OPC programme manager characterized it as “an interesting tool to make available to all trainers and CDDs”. Teachers reported that the passport was “very useful”, “explicit”, “pedagogical”, and “easy to understand”, and emphasized its dual role as both a training and motivational tool. Its uptake clarified distribution protocols, improved cascade training, and reinforced frontline engagement. These findings were consistent across groups. CDDs highlighted increased confidence and clarity in their roles, community members reported improved trust and understanding, and trainers emphasized the practical value of the passport in delivering consistent and structured training. Strong demand for additional copies and versions in both French and Chadian Arabic underscored its perceived value and potential for broader scale-up.

Provincial and departmental health authorities and implementing partners likewise recognized the passport as a practical, concise reference for community and school-based sensitization activities, supporting outreach and health education. Health center staff noted its effectiveness in improving community awareness of NTDs, particularly among parents, while also enhancing professional recognition of CDDs.

Nonetheless, several challenges were noted. The length of the passport [as originally tested] increased printing costs, photographs for CDD identification could not be included due to resource constraints, and availability was limited to a single language version. Feedback also indicated the need for more detailed information on disease transmission vectors. Recommended refinements include condensing content to essential elements, adding visual aids such as vector images, producing bilingual editions (French and Arabic), including CDD photographs, and clarifying verification and stamping procedures at national or sub-national levels. Given cost concerns, it was suggested that distribution be prioritized in districts with persistent programmatic challenges.

Finally, broader discussions during the study highlighted the evolving recognition of CDDs as CHWs within national health policy. While such recognition is strengthening, important gaps remain, particularly for new CDDs, teachers participating in school-based campaigns, and other influential community volunteers. Although national policies in Chad increasingly support CHW recruitment and recognition, maintaining these resources in the context of disease elimination represents an ongoing challenge.

## 5 Discussion

### 5.1 Strengths and limitations of the case study

The introduction of the CDD passport in Chad represents a programmatic innovation addressing key barriers to volunteer motivation, knowledge, and recognition. Beyond its function as a training aid, the passport served as a hybrid tool combining practical guidance, continuity of learning, and symbolic recognition. As an official, named record of training, it strengthened CDDs’ sense of legitimacy and professional identity, reinforcing confidence during community engagement. Consolidating core technical content, key messages, and role clarification into a single portable resource reduced reliance on fragmented training and social and behaviour change (SBC) materials—an ongoing challenge in cascade training and MDA delivery. Embedded within routine programme processes, the passport improved capacity and morale, bridged knowledge gaps, and strengthened trust at the community–health system interface. These findings align with programme feedback that emphasizes the importance of recognition, role clarity, and integrated support for sustaining volunteer health workforces. The passport also prompted government reflection on gaps in CHW policy, underscoring the need for clearer and more harmonized frameworks for sustainable integration.

Limitations include the study’s focus on Chad and the early stage of passport implementation. Design and roll-out were shaped by contextual and operational constraints, including production costs, printing and distribution challenges, language mismatches with CDD literacy, and variability in training quality and supervision. Although widely adopted as a training and job aid, effective use depended on facilitator capacity, availability of complementary materials, and the broader organizational context. Key enablers included strong co-design with government and programme staff, integration into routine training and MDA processes, and the passport’s dual role as a job aid and recognition tool. Future iterations should streamline content, expand visual aids, and address language and cost barriers to enhance accessibility and scalability. Longer-term evaluation is needed to assess impacts on coverage, volunteer retention, health outcomes, and cost-effectiveness, as well as institutionalization within national CHW policies. Planned roll-out in additional countries provides opportunities for comparative analysis of contextual adaptability, implementation readiness, and policy alignment.

### 5.2 Implications for policy and practice

The evaluation of this program innovation highlights critical challenges and opportunities concerning the formal recognition and support of volunteer CHWs, particularly CDDs, in NTD programs and broader health systems. The findings underscore the persistent policy ambiguity that inhibits the optimal integration of these volunteers into UHC frameworks, despite their indispensable contribution to MDA campaigns and community health more broadly.

This initiative revealed structural issues within current NTD and CHW policies at national and global levels, particularly the tendency to view CDDs narrowly as MDA implementers rather than as generalist CHWs with diverse roles throughout the year. This narrow focus obscures their broader contributions—such as community mobilization, health education, morbidity management, and surveillance activities—and can reduce community understanding and health system support. The case study from Chad supports prior calls for revisiting and expanding CHW definitions and recognition policies to better capture this diversity and complexity.

Donor and NGO-driven program demands often diverge from national policy intentions, sometimes undermining sustainable volunteer engagement by imposing inflexible tasks or insufficient support. The CDD passport’s co-creation approach with the Ministry of Health demonstrates the value of inclusive, locally adapted solutions that respect community dynamics and contextual realities, including linguistic diversity, population distribution, and community structure.

Sustaining volunteer CHWs in the evolving NTD landscape—where treatment needs may decline but become concentrated in harder-to-reach populations (12)—requires policy shifts that formalize volunteer roles, enhance incentives, improve supervision, and better integrate CHWs within decentralized health governance. This aligns with WHO’s emerging frameworks endorsing community health workforce strengthening as fundamental to UHC (13). Messaging around volunteer recognition has implications beyond NTDs, informing policy discussions for integrated community health.

Future iterations of the passport have begun to explore hybrid formats that integrate digital components. In more recent versions deployed in other country contexts, a QR code has been introduced on the back of the passport, allowing CDDs with access to mobile devices to scan and retrieve additional content. This includes expanded guidance, refresher materials, and opportunities for ongoing learning beyond initial training. Such approaches offer potential to support more dynamic and updatable training content, enable interaction with learning platforms, and strengthen supervision and engagement over time. While connectivity and device access remain uneven in many implementation settings, the progressive expansion of mobile networks presents opportunities to gradually integrate digital enhancements into the passport model, improving its flexibility, scalability, and long-term relevance.

## 6 Conclusion

The CDD passport initiative in Chad demonstrates the value of formal recognition and structured training for volunteer community health workers engaged in NTD control. It reinforces the significant contributions of CDDs as integral members of the health workforce and creates momentum for policy reforms to better integrate volunteers into national health systems. Experiences from the CDD passport rollout in Chad offer practical guidance for WHO and policymakers to align CDD selection, training, supervision, and support with national universal health coverage frameworks.

By bridging programmatic innovation and policy dialogue, the passport supports sustained volunteer engagement and strengthens progress toward universal health coverage. In spite of these numerous advantages to improving the motivation of the CDD, the passport alone will not resolve the controversy concerning the role, motivation, management and recognition of the community drug distributor as a significant cadre for the attainment of universal health coverage.

## Data Availability

The raw data supporting the conclusions of this article will be made available by the authors, without undue reservation.
