# Supplementary material for: The community drug distributor passport: a case study of training and recognition in Chad
Source: Front Public Health. 2026 Sep 3;14:1879806. doi: 10.3389/fpubh.2026.1879806 (PMC13581789; doi:10.3389/fpubh.2026.1879806)
Supplement: Supplementary file 1 [file Supplementary_file_1.pdf]

RÉPUBLIQUE DU TCHAD
MINISTÈRE DE LA SANTÉ PUBLIQUE

Passeport de l'agent distributeur de médicaments

OPC EMPOWERING THROUGH SIGHT

Sightsavers

© Sightsavers, Ministère de la Santé du Tchad et OPC, 2026. Tous droits réservés.

Le Passeport de l'agent distributeur de médicaments a été élaboré par Sightsavers en partenariat avec le ministère de la Santé du Tchad et l'OPC. Son contenu est mis à disposition afin de faciliter l'apprentissage et la mise en œuvre des programmes. Toute personne souhaitant reproduire, adapter ou redistribuer tout ou partie de ce document doit en citer la source et obtenir l'autorisation écrite préalable de Sightsavers.

Page de contenu

1. Les informations d'identification de l'agent ______ 4

2. Les MTNs traitees au Tchad ______ 8

3. Une bonne preparation d'une distribution des medicaments ______ 11

4. Les etapes de la distribution des medicaments ______ 16

5. Les effets secondaires suite a la prise des medicaments ______ 29

6. Informations sur les maladies traitees ______ 32

7. Les messages cles a difuser ______ 36

8. Les contacts d'urgence ______ 42

1. Les informations d'identification de l'agent

Ce passeport est un outil de travail créé dans le but de faciliter l'identification des agents impliqués dans les campagnes de distribution.

Photo

Nom : ______________________

Prénom : ______________________

Date d'émission : ______________________

Formé :

- ☐ Oui
- ☐ Non

Signature : ______________________________

Cachet : ______________________________

Informations supplémentaires

Fonction:________________________________

Lieu d'intervention

District sanitaire :________________________

De sant :________________________________

Zone d'intervention :______________________

Participation à des campagnes de distribution de masse

Nom de la maladie	Date de la formation	Rôle
Onchocerchose	______<br>______<br>______<br>______<br>______	______<br>______<br>______<br>______<br>______
Filariose lymphatique	______<br>______<br>______<br>______<br>______	______<br>______<br>______<br>______<br>______

Nom de la maladie	Date de la formation	Rôle
Geohelminthiases	______<br>______<br>______<br>______<br>______	______<br>______<br>______<br>______<br>______
Schistosomiase ou bilharziose	______<br>______<br>______<br>______<br>______	______<br>______<br>______<br>______<br>______

2. Les MTNs traitees au Tchad

Au Chad, les Maladies Tropicales Négligées (MTN) représentent un problème important pour la sante de la population. Les MTN sont un groupe de maladies infectieuses qui affectent principalement les populations qui n'observent pas les bonnes pratiques d'eau, d'hygiène et d'assainissement recommandées.

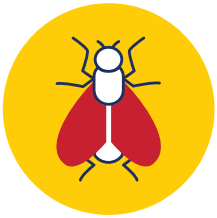

L’onchocerchose : également connue sous le nom de “cécité des rivières”, elle est causée par des parasites qui sont transmis par des mouches noires. La maladie affecte les yeux et peut entraîner une perte de la vue permanente si elle n’est pas traitée

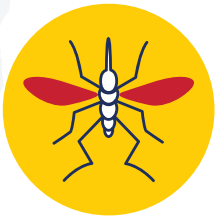

La filariose lymphatique :
elle est causée par des parasites transmis par des moustiques. La maladie provoque un gonflement des membres, du scrotum ou des seins, entraînant une incapacité physique et d’autres complications

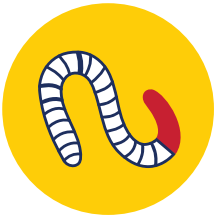

Geohelminthiases : Les geohelminthiases ou vers intestinaux sont une infection causée par les parasites intestinaux. Les vers vivent dans les intestins humains et affectent la nutrition en empêchant l'absorption des nutriments. Les symptômes comprennent la douleur intestinale, la malnutrition, le ralentissement de la croissance et du développement physique

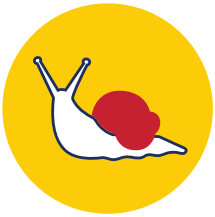

La schistosomiase ou la bilharziose : c'est une maladie parasitaire causée par des vers plats qui se propagent dans l'eau contaminée. Elle affecte principalement les personnes qui sont en contact avec des eaux douces infestées comme les rivières et les lacs

3. Une bonne preparation d'une distribution des medicaments

Le rôle du distributeur et les procédures de distribution pendant la distribution

En tant qu'agent distributeur de médicaments, ton rôle est de :

- Accueillir les bénéficiaires : saluer les bénéficiaires et leur expliquer l'importance de prendre le medicament et les effets secondaires possibles
- Verifier les informations de base : s'assurer pour chaque enfant qu'il n'est pas dans une situation d'une personne qui ne doit pas prendre le médicament
- Distribuer correctement du médicament

Voici la liste des materiaux a avoir pour realiser une bonne distribution des medicaments

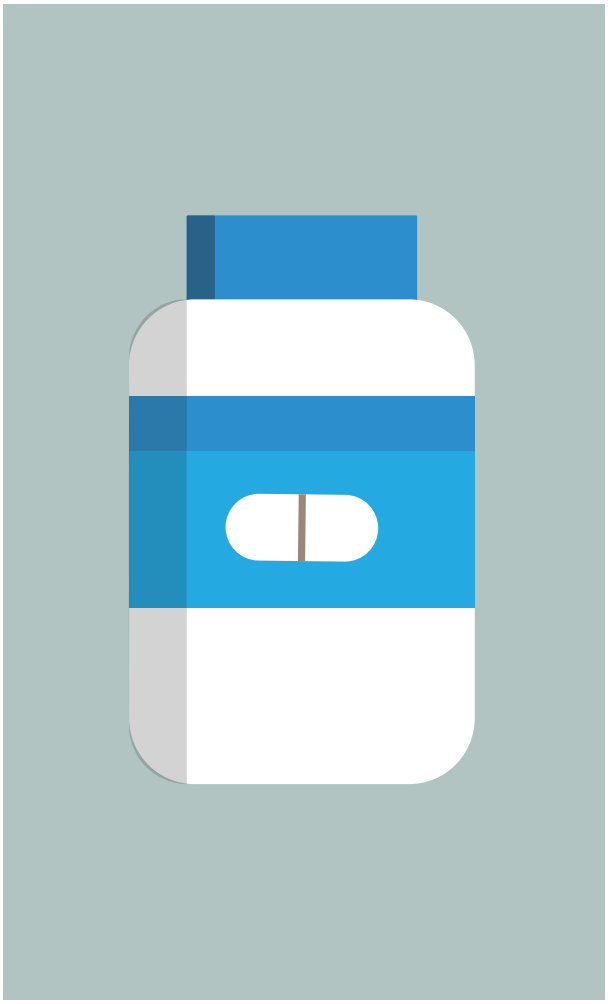

Boite de médicaments (Le conserver dans son emballage d'origine et bien fermé pour le protéger de la lumière du soleil)

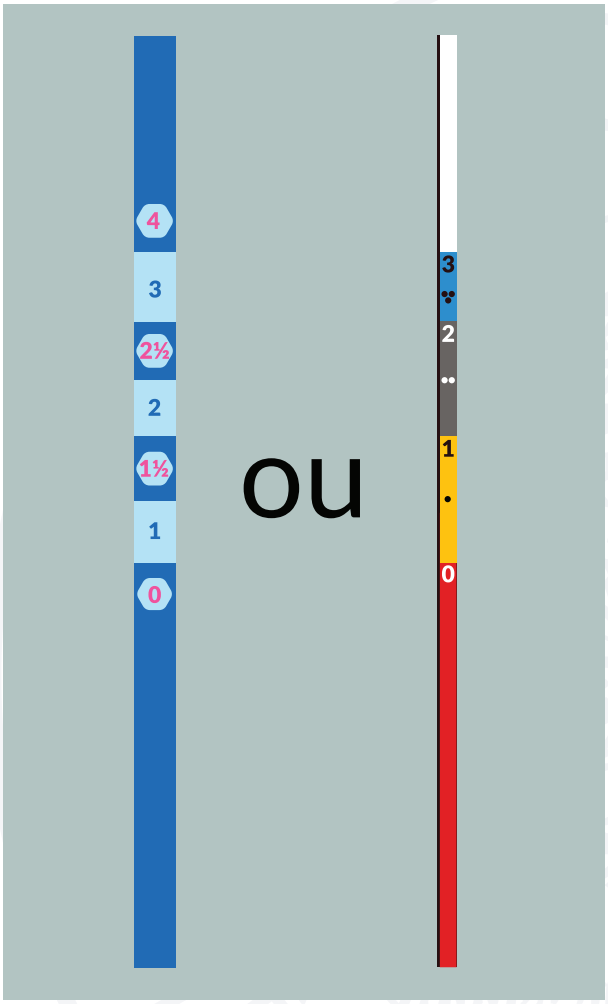

La toise pour mesurer la dose

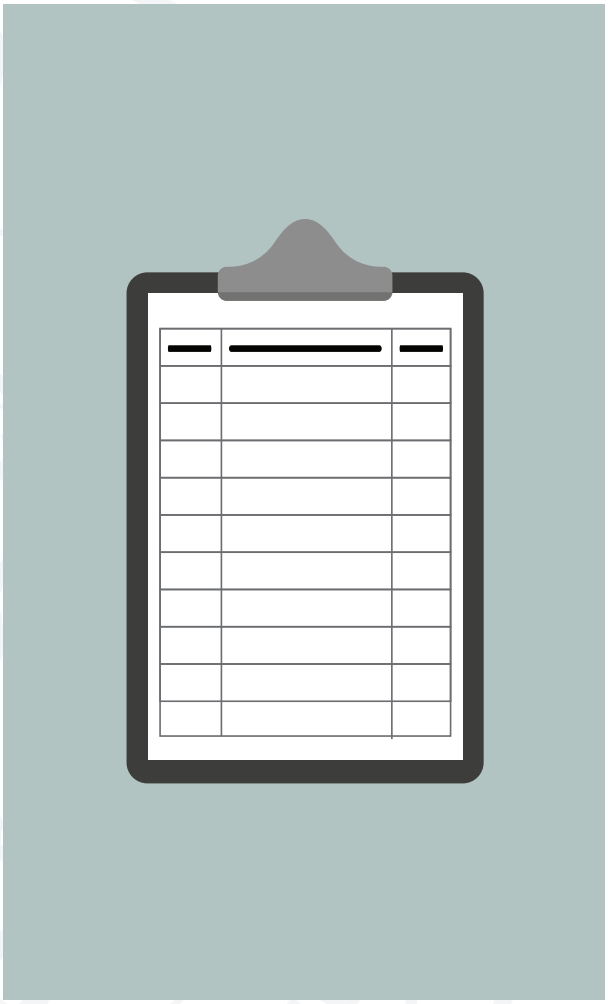

Le registre pour
enregistrer
les informations

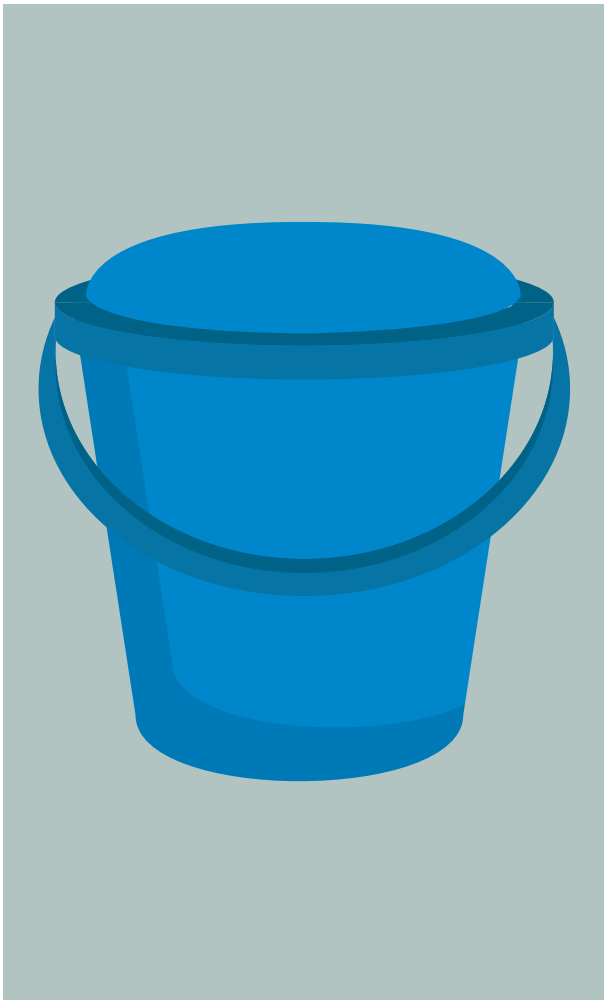

Un seau propre

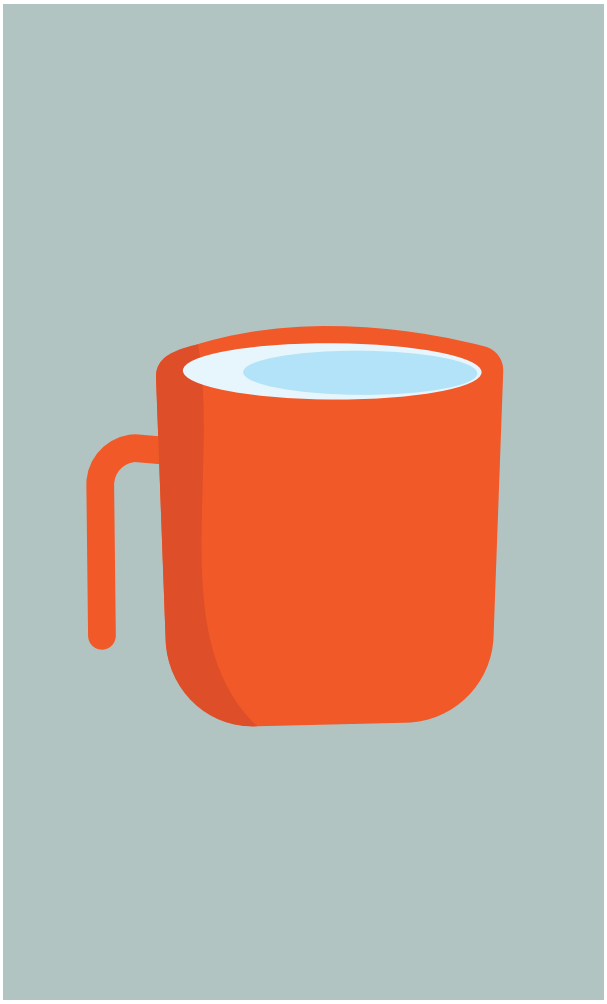

De l’eau potable

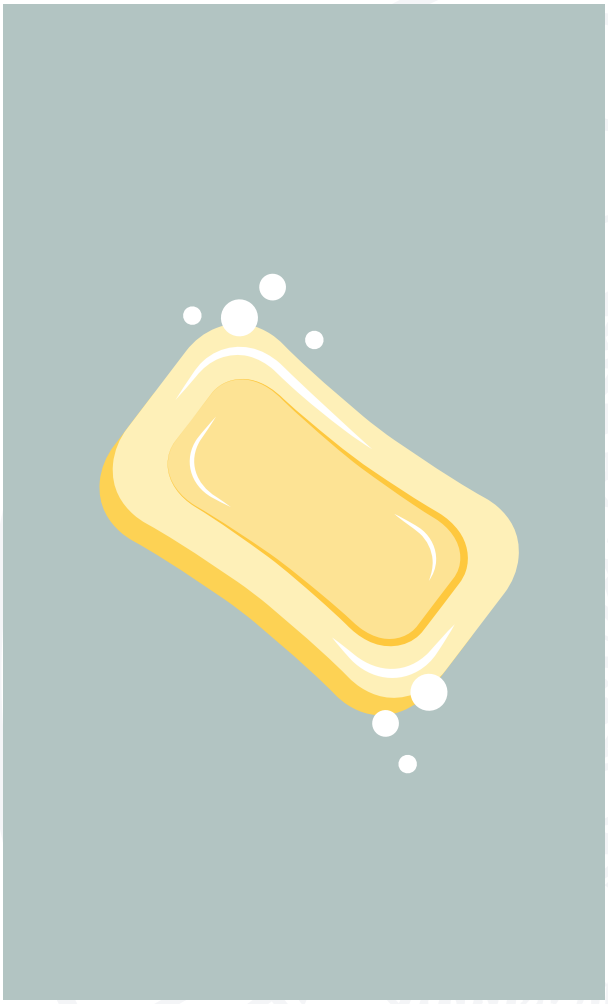

Du savon

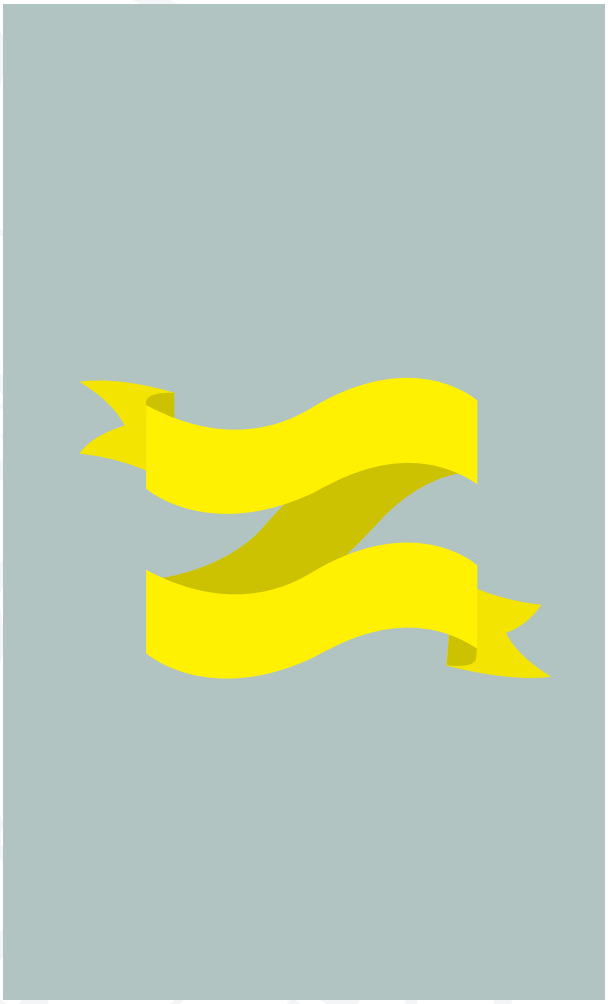

Un ruban a attacher
au poignet de l’enfant

4. Les etapes de la distribution des medicaments

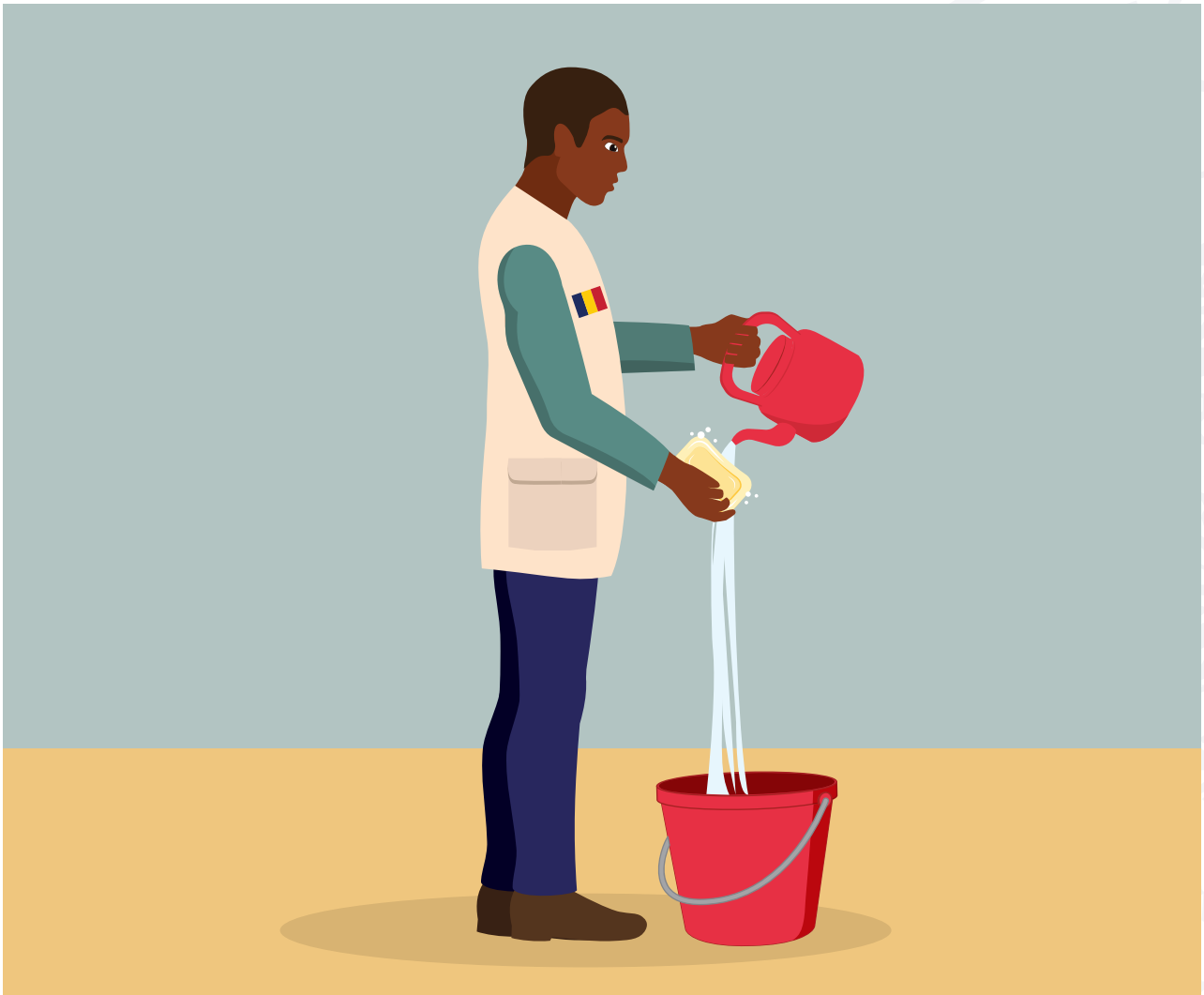

1. Se laver les mains avec de l' eau et du savonavant chaque séance de distribution

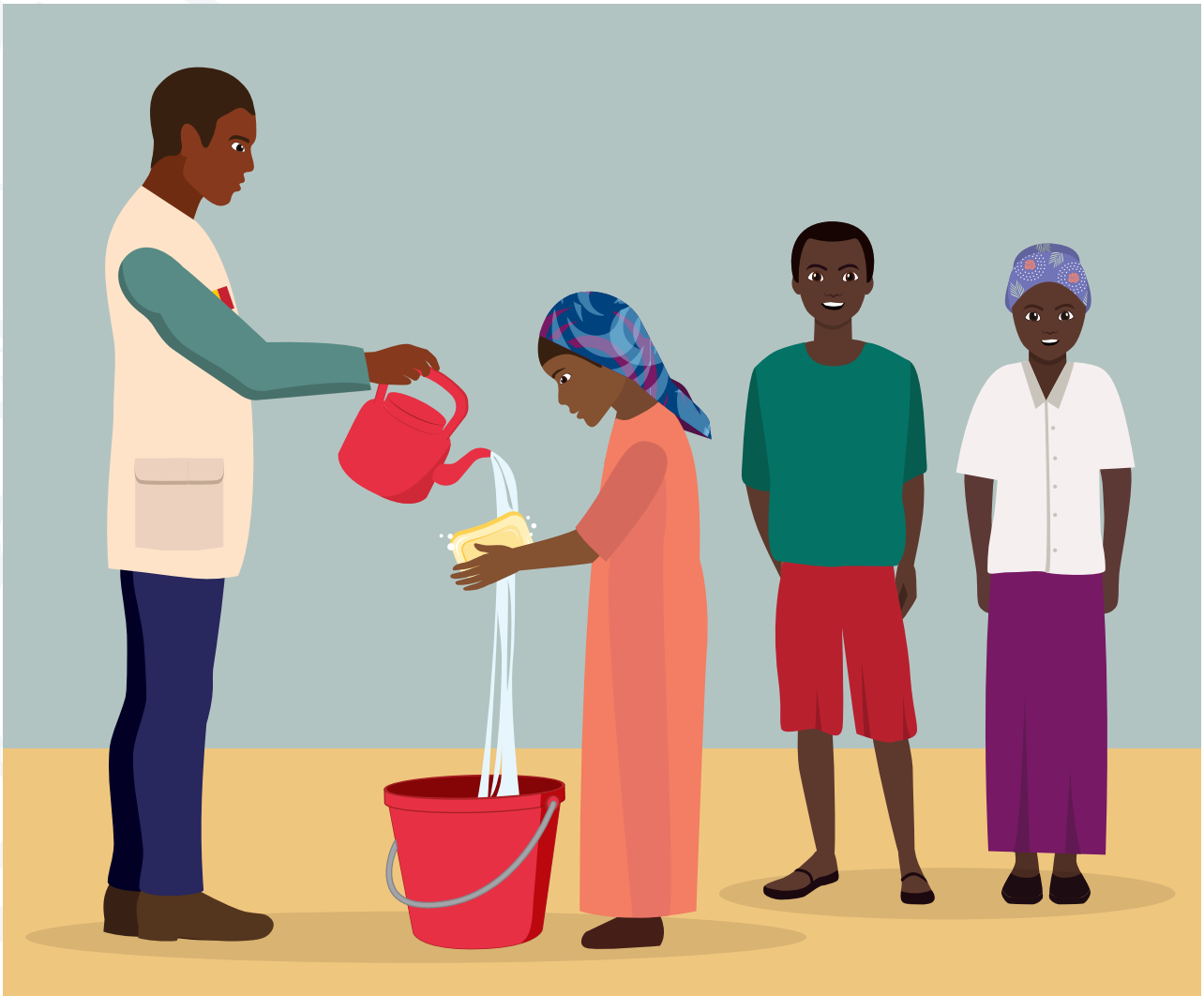

2. Demander aux enfants de se laver les mains avec de l'eau et du savon avant de toucher le médicament

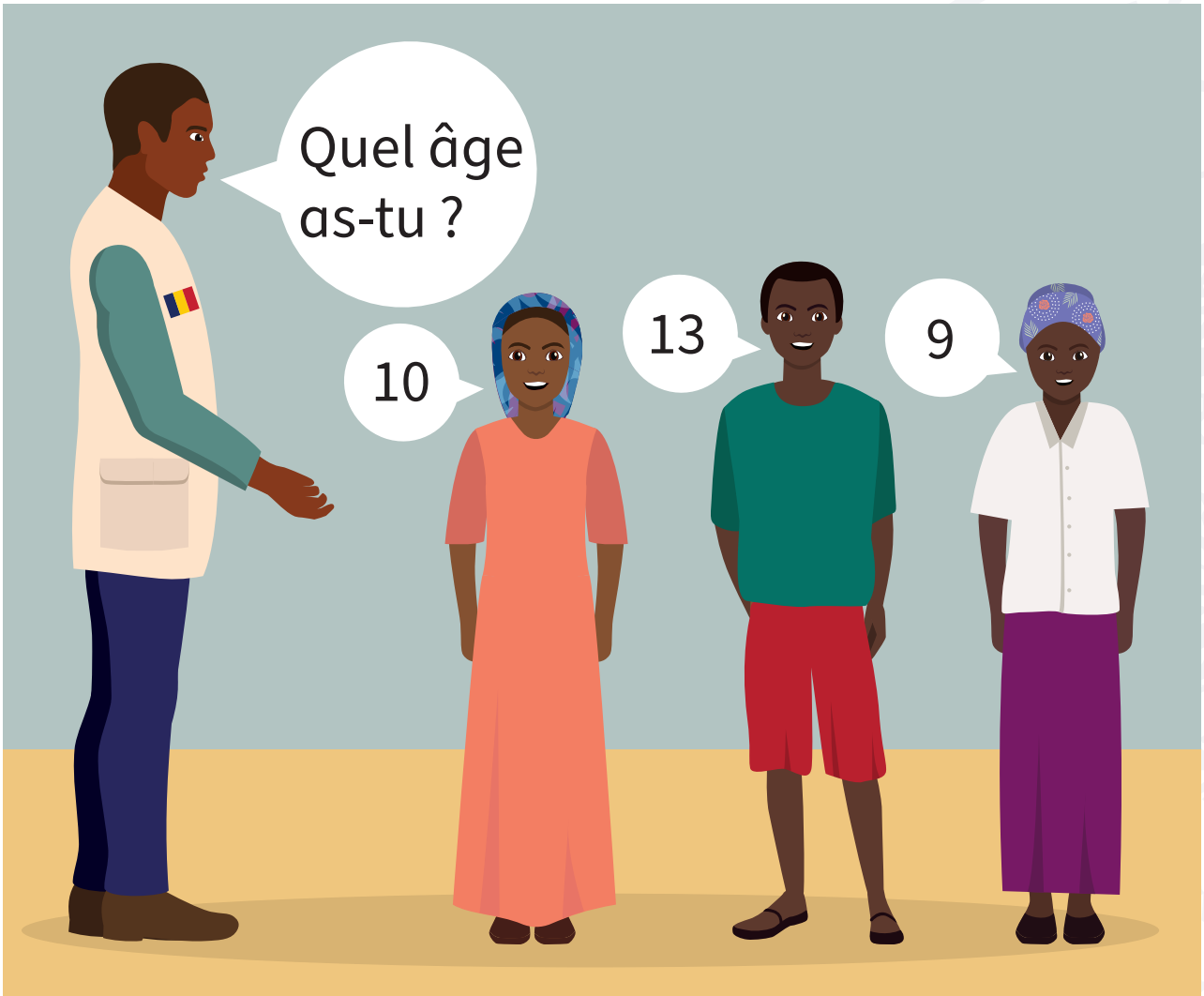

3. Vérifier l'âge de l'enfant. S'il a moins de 5 ans, tu ne dois pas lui donner le médicament et s'il a plus de 14 ans tu ne dois pas aussi lui donner le médicament

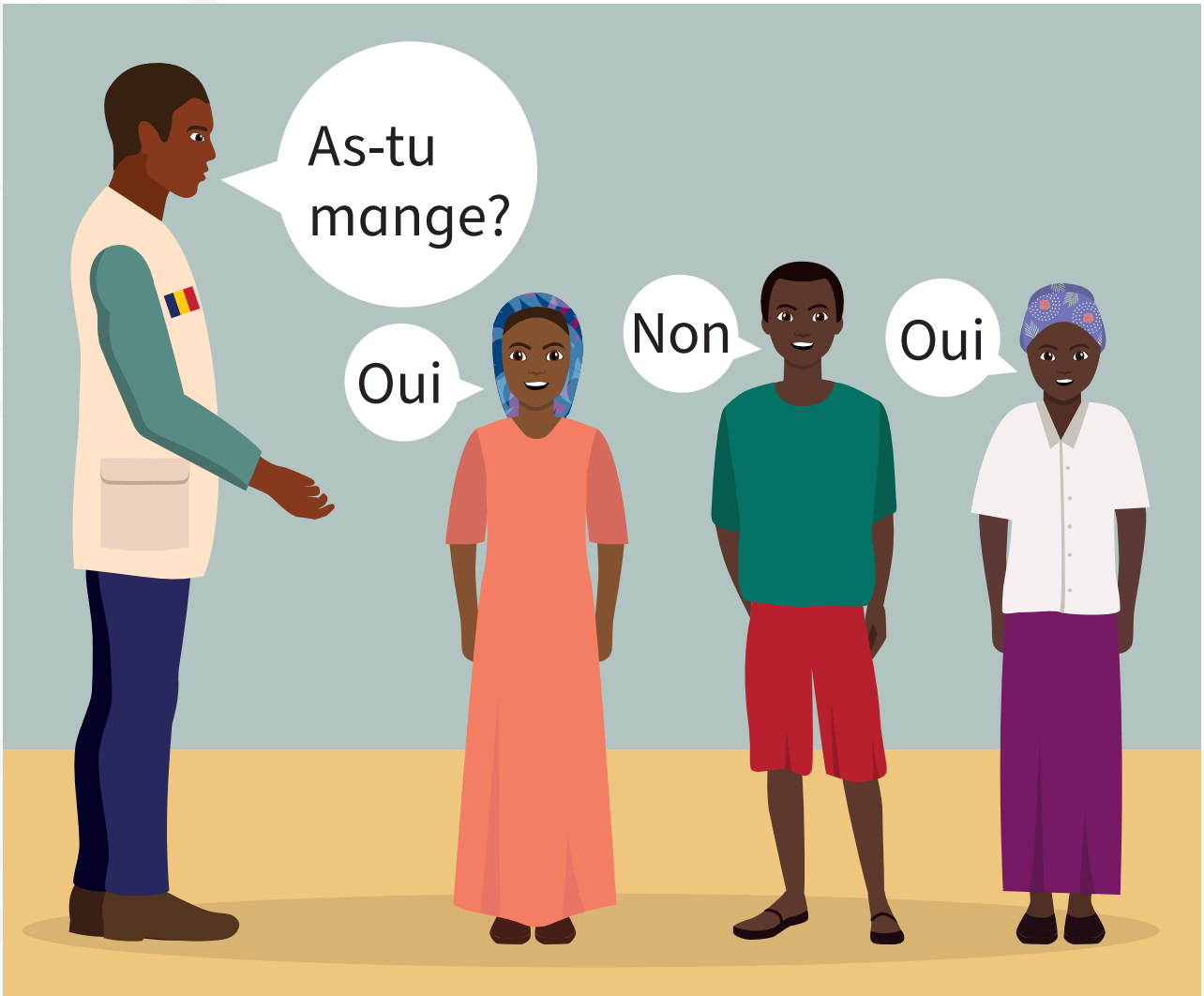

4. Verifier si l'enfant a mangé. Si non lui demander d'aller manger quelque chose et revenir pour prendre le médicament

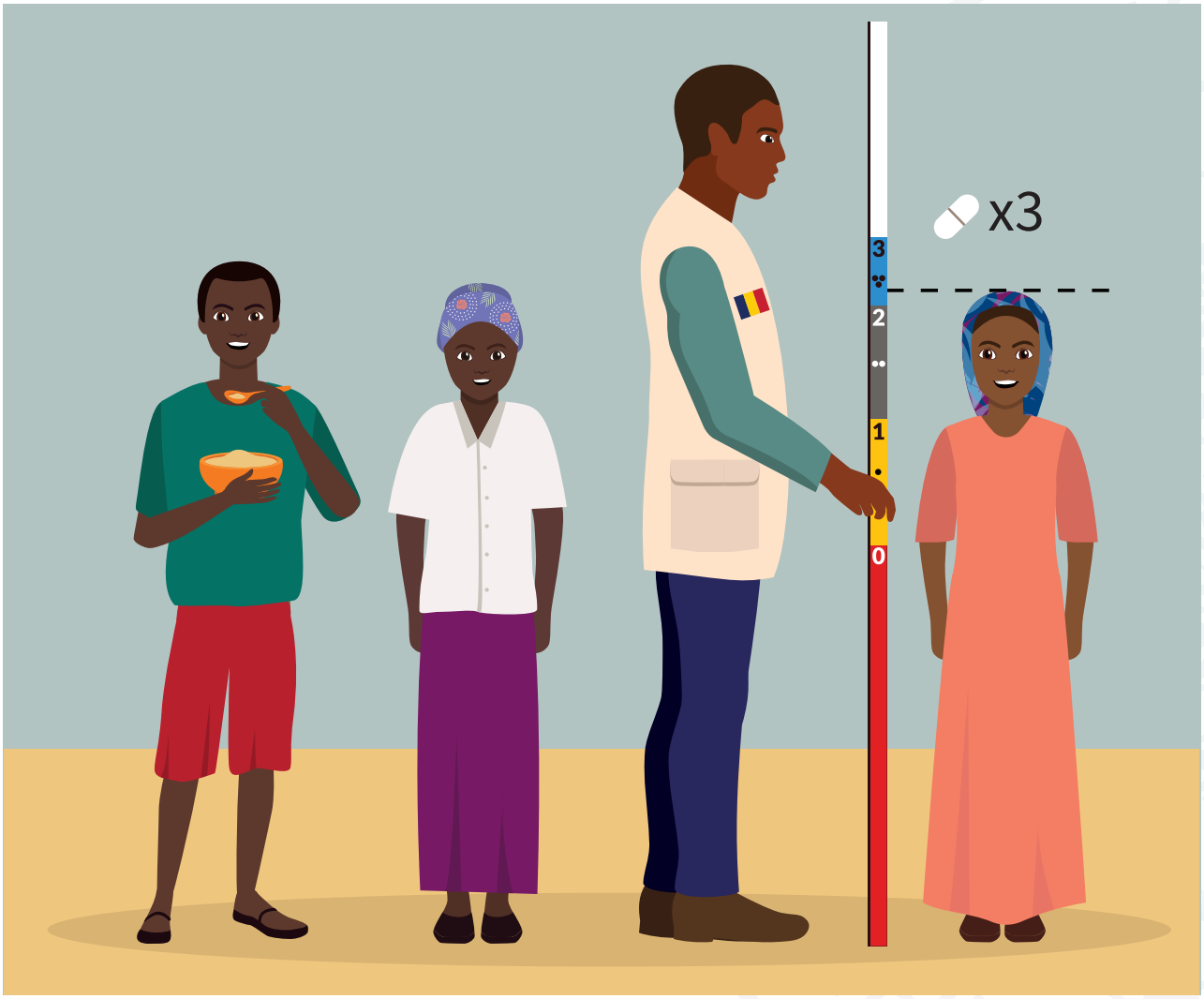

5. Avec la toise, tu dois mesurer l'enfant et lui donner la dose qui correspond à sa taille

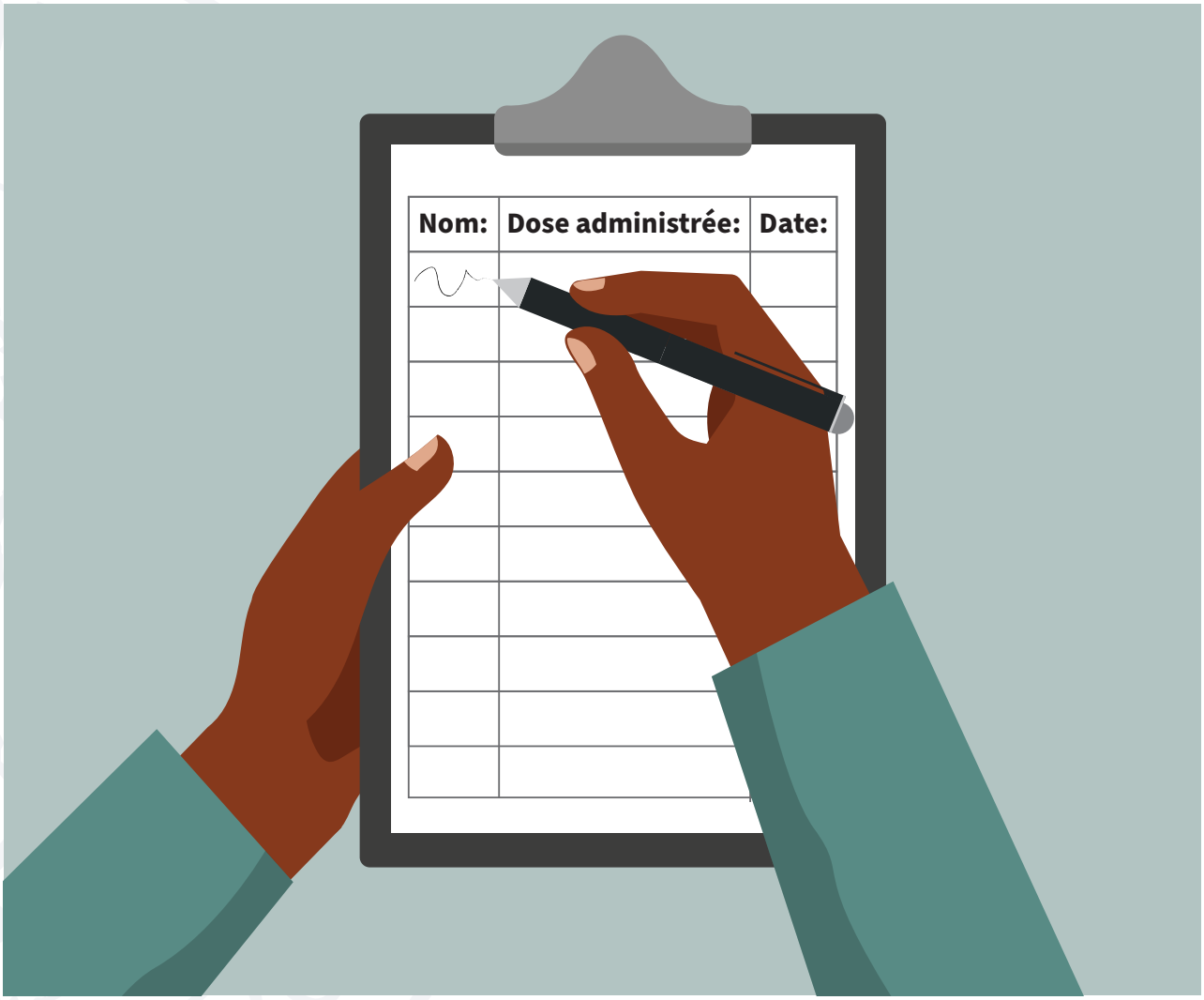

6. Inscrire sur ton registre les informations sur l'enfant (son nom, la dose qui lui a été donnée et la date d'administration)

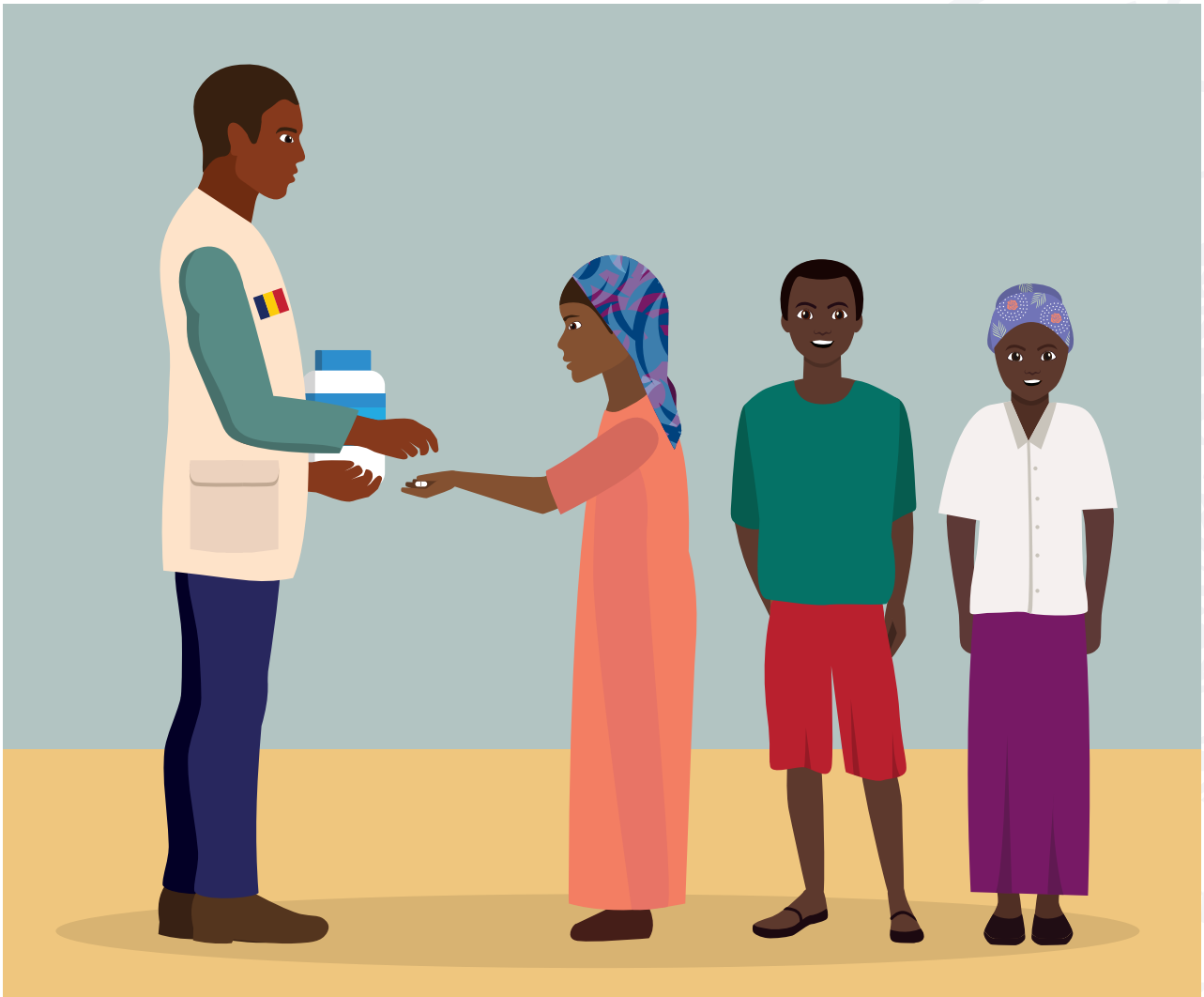

7. Donner le médicament à l'enfant

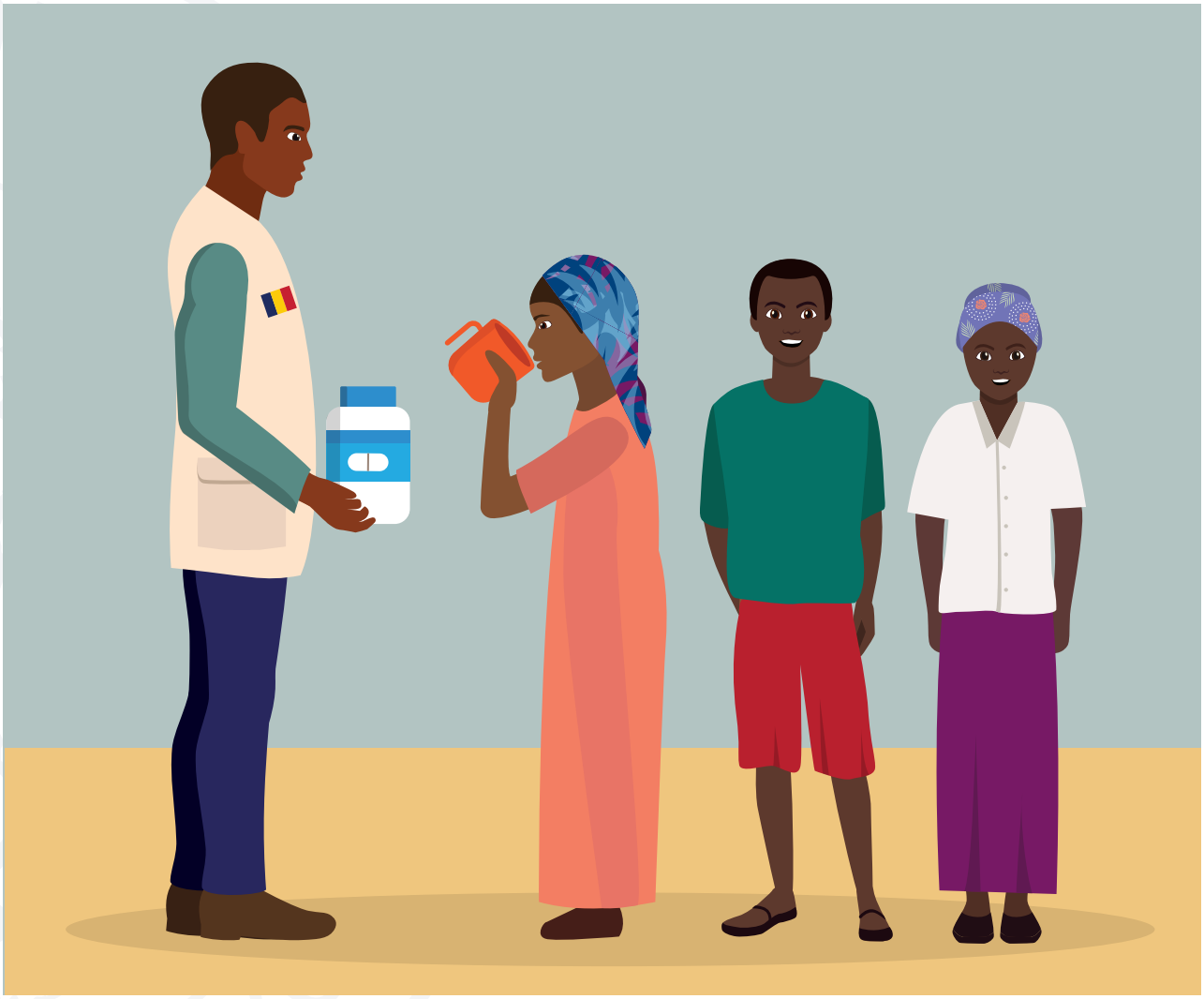

8. S’assurer qu’il avale complètement le medicament avec de l’eau

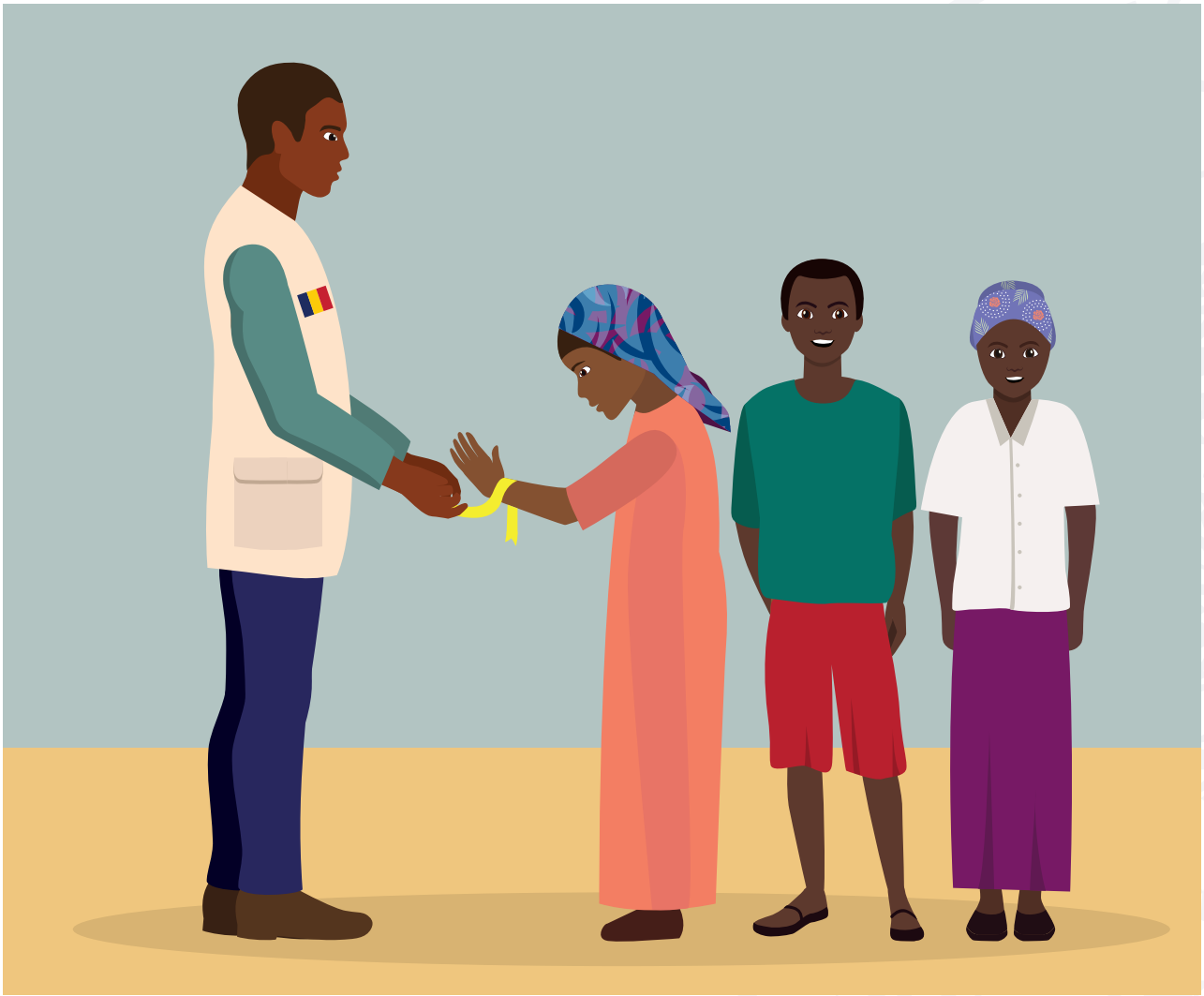

9. Attacher le ruban autour du poignet de l'enfant

Attention : Voici les différentes situations de personne a qui il ne faut pas donner le médicament

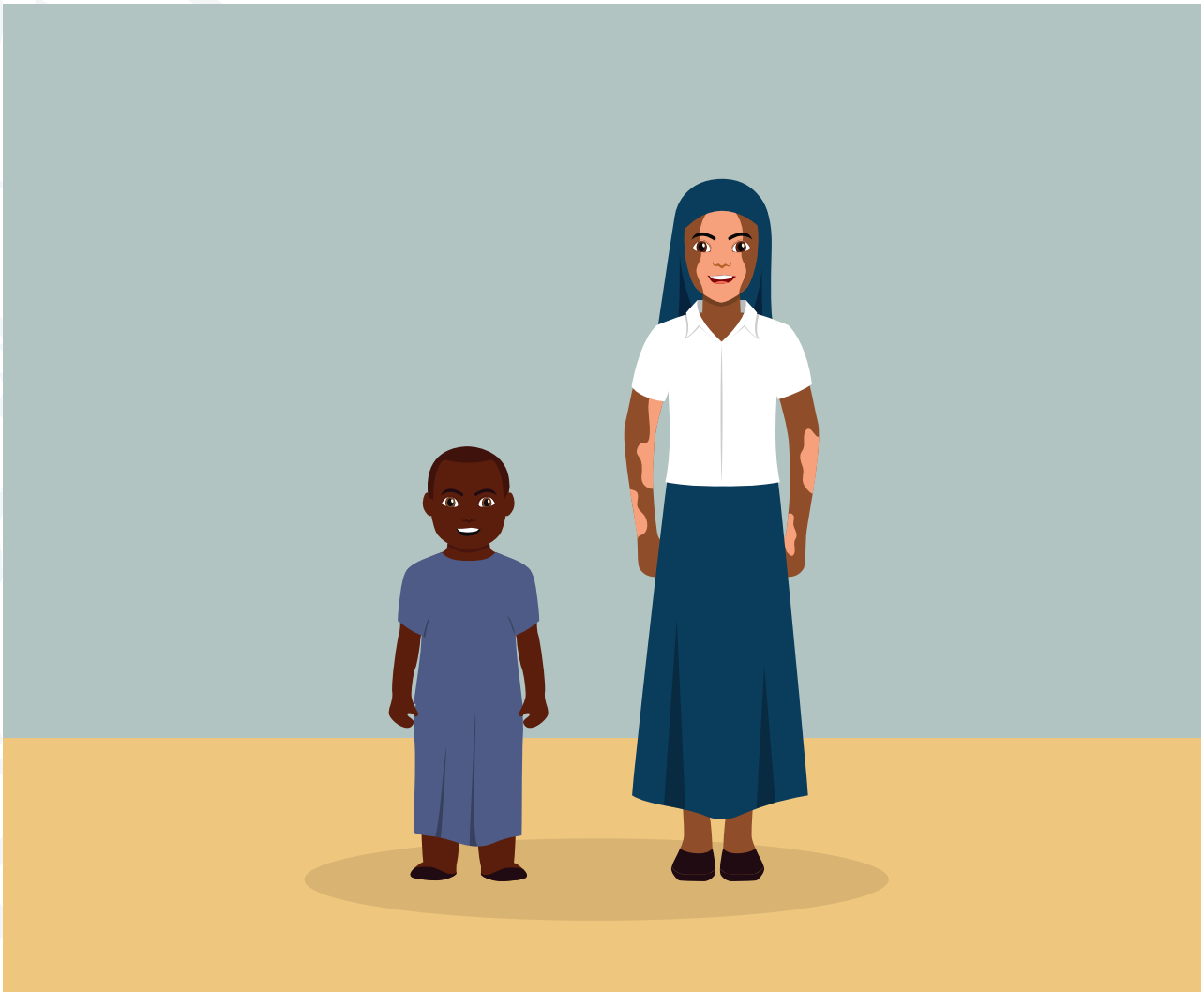

- Un garçon ou une fille de moins de 5 ans et de plus de 14 ans

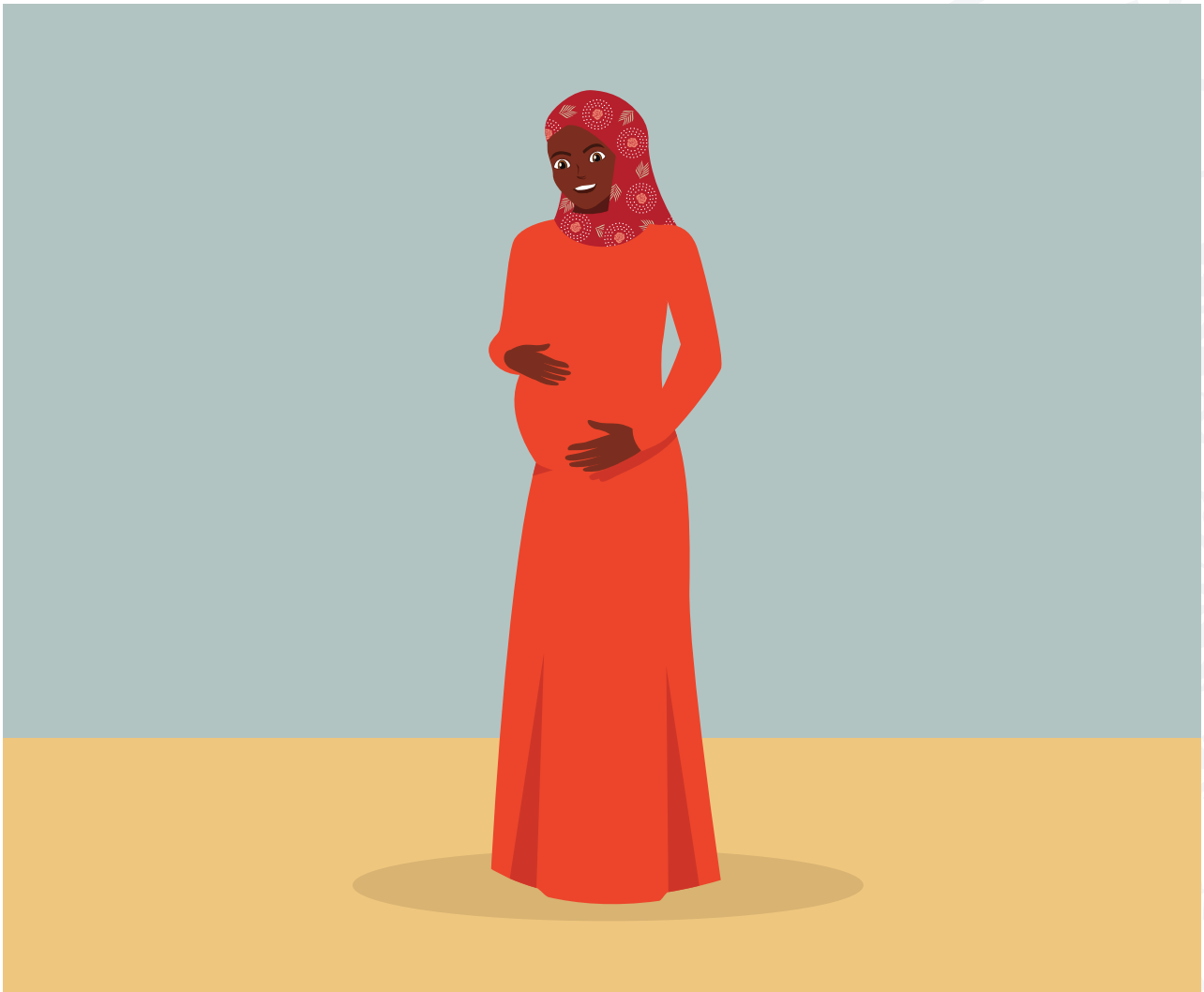

- Une fille qui est enceinte

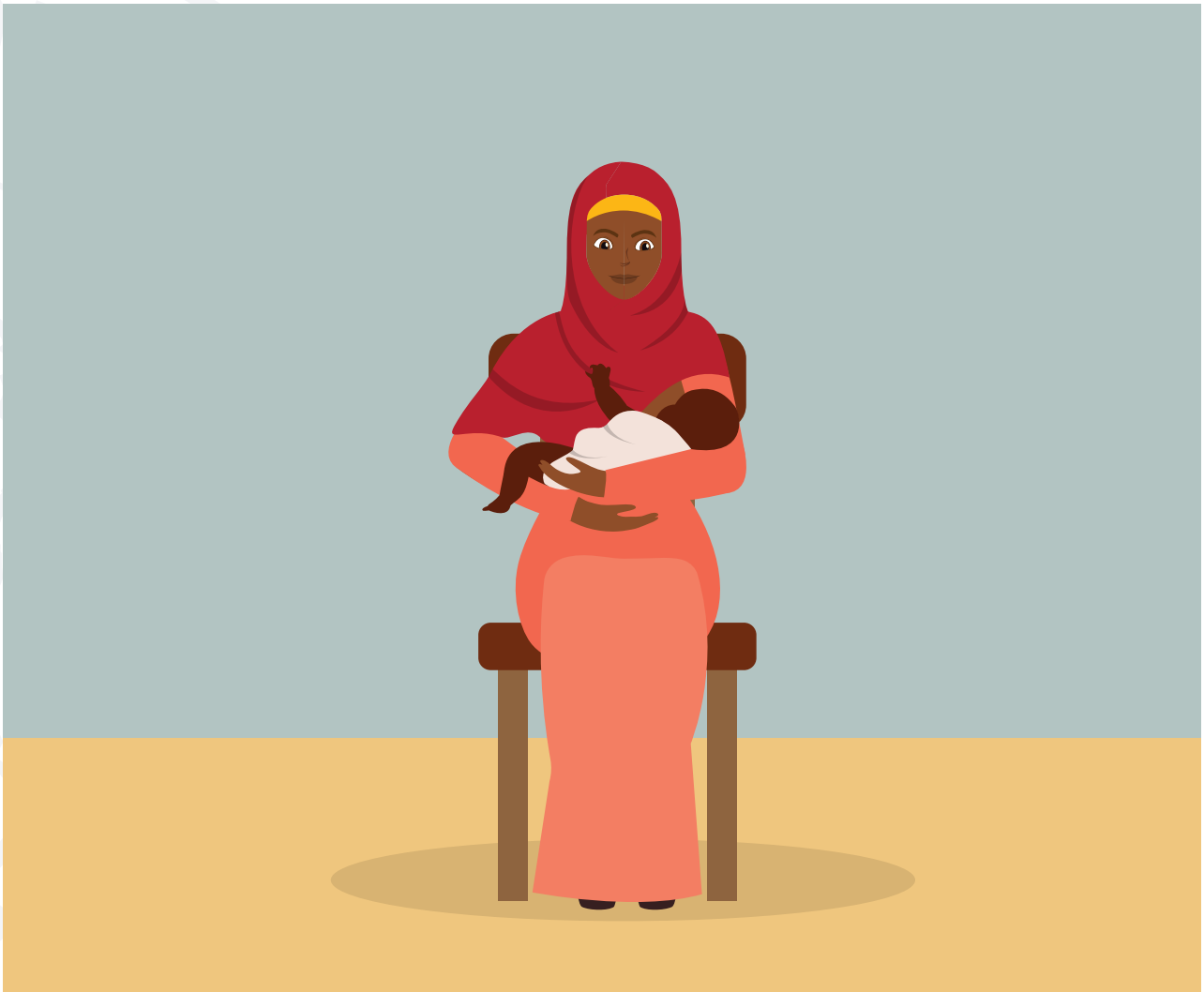

- Une fille qui allaite un enfant

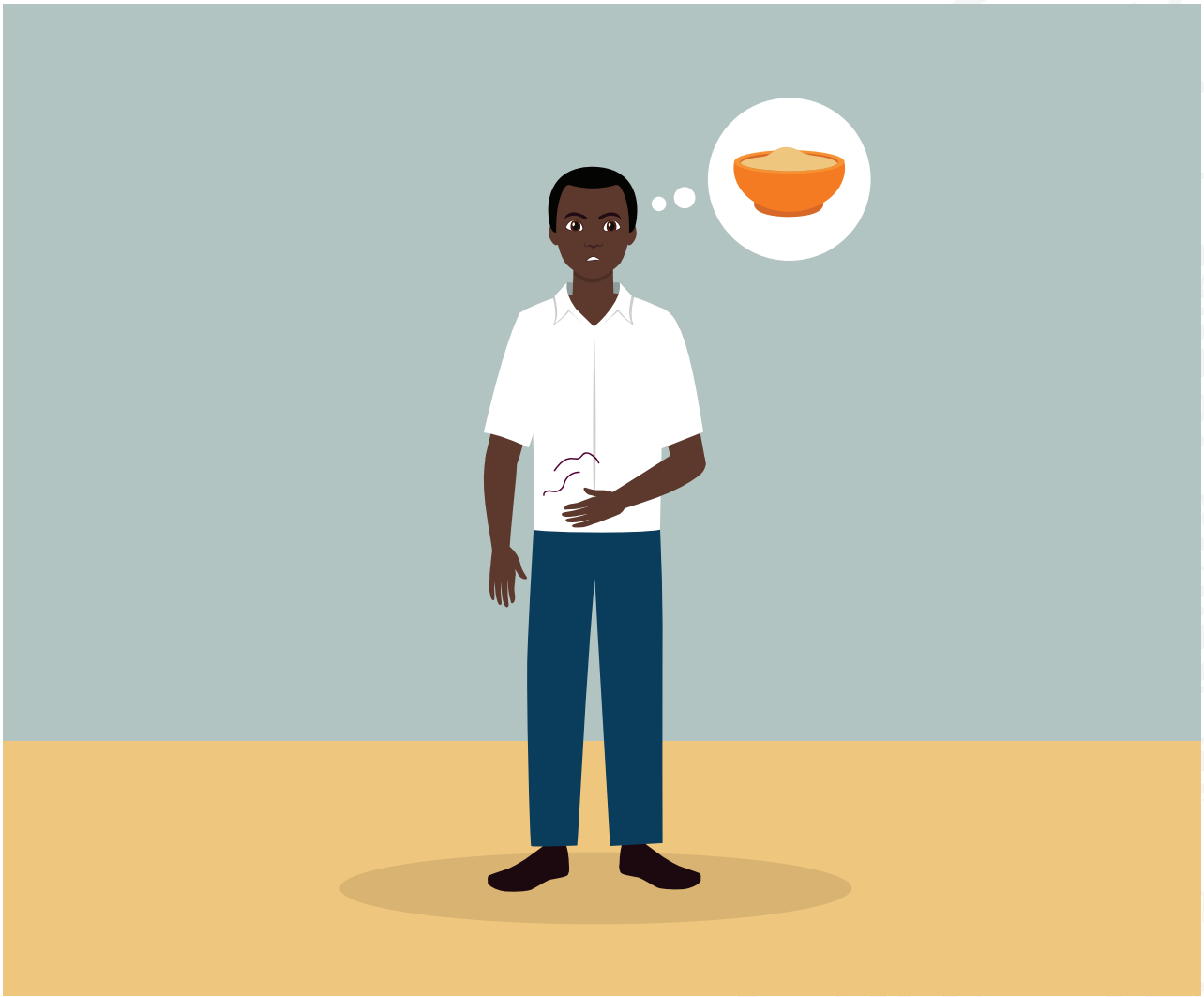

- Une personne qui n’a pas mangé

5. Les effets secondaires suite a la prise des medicaments

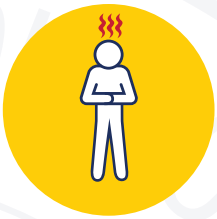

Les nausées

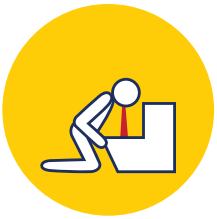

Les vomissements

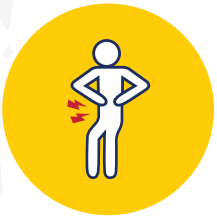

Douleurs abdominales

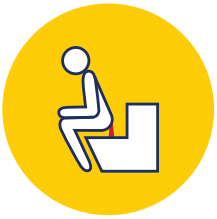

Les diarrhées

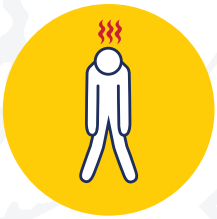

Fatigue

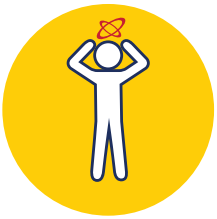

Des vertiges

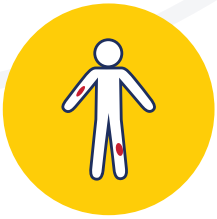

Des rougeurs sur la peau

Comment prévenir et gérer un effet secondaire survenu a la suite de la prise du médicament Praziquantel ?

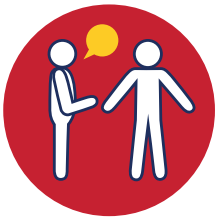

Informer : avant de donner le médicament il faut expliquer sur les effets secondaires possibles et expliquer que ces effets généralement ne durent pas trop longtemps et sont sans danger

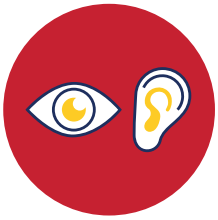

Sureveiller : vous devez encourager les enfants a signaler tout effet secondaire qu'ils pourraient ressentir après avoir pris le praziquantel

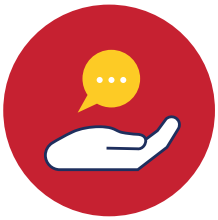

Conseiller et soutenir : A la suite de la prise du praziquantel, si un enfant ressent des effets secondaires légers tels que des maux d'estomac et de la fatigue, vous pouvez lui conseiller de prendre une solution sucrée-salée et de prendre un peu de repos

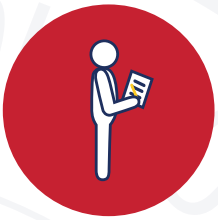

Référer : si un enfant présente des effets secondaires plus graves, vous devez automatiquement le référer vers un professionnel de santé

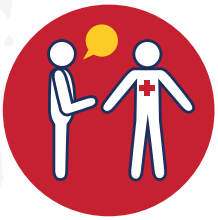

Documenter : noter sur les registre, les cas d'effets secondaires rencontrés sur le terrain suite à la prise du praziquantel par un enfant

6. Informations sur les maladies traitees

C'est quoi cette maladie ?

La schistosomiase ou Bilharziose est une maladie causée par des vers plats qui vivent dans l'intestin ou dans la vessie de l'homme/ femme infecté. On distingue: la bilharziose urinaire et la bilharziose intestinale.

Comment attrape-t-on cette maladie ?

Une personne parasitée contamine l'eau en faisant les selles ou en urinant dans l'environnement ou dans l'eau. Lorsqu'une personne en santé vient ensuite se laver, faire la lessive ou la vaisselle, la pêche ou la culture du riz dans la zone infestée, elle peut attraper la maladie.

Comment peut-on reconnaître la maladie ?

- Douleurs abdominales
- Sang dans les selles
- Toux
- Fatigue
- Migraine
- Éruption cutanée
- Douleurs corporelles
- Anémie
- Sang dans les urines
- Douleur ou difficultés pour uriner

Comment attrape-t-on cette maladie ?

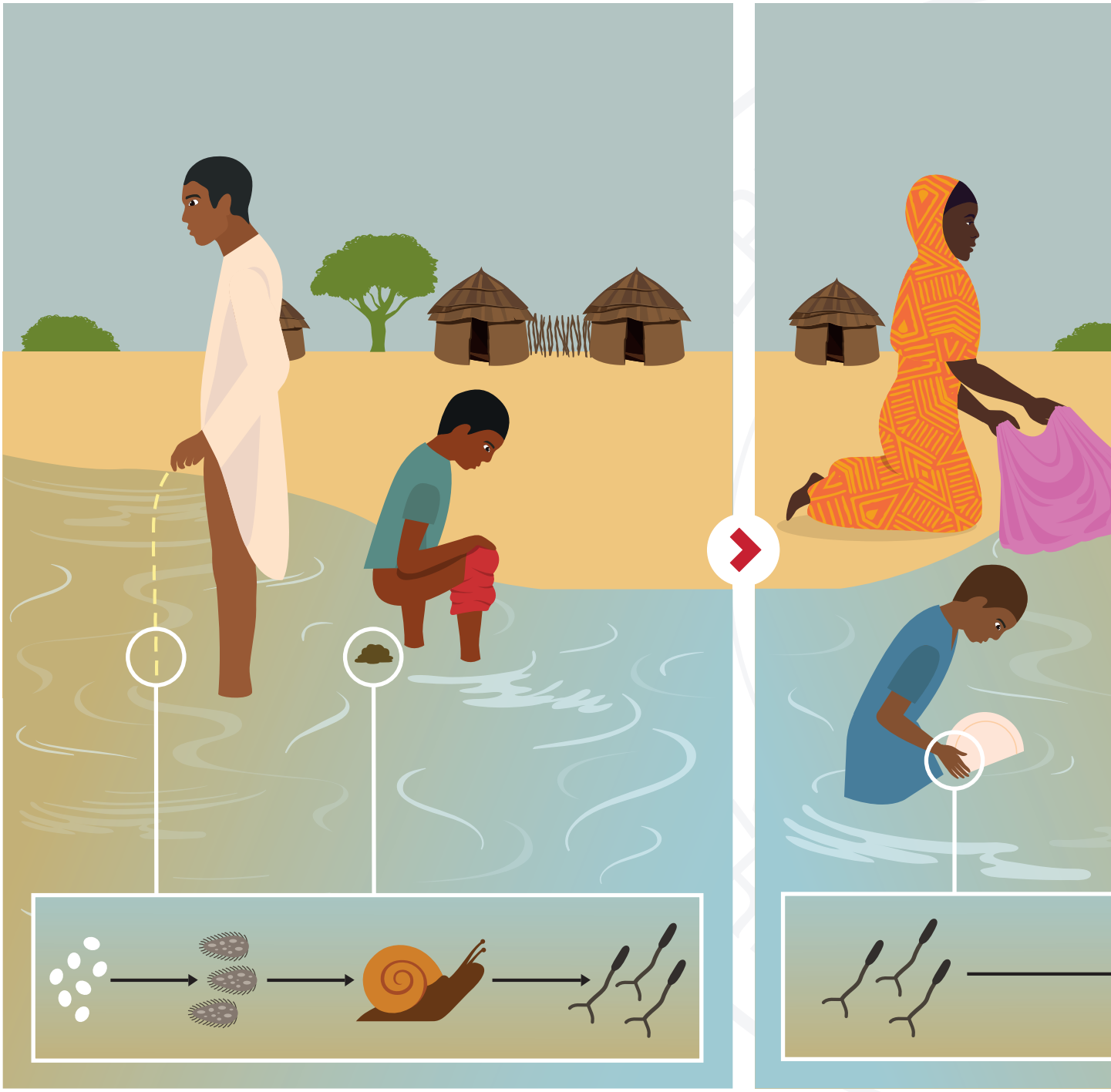

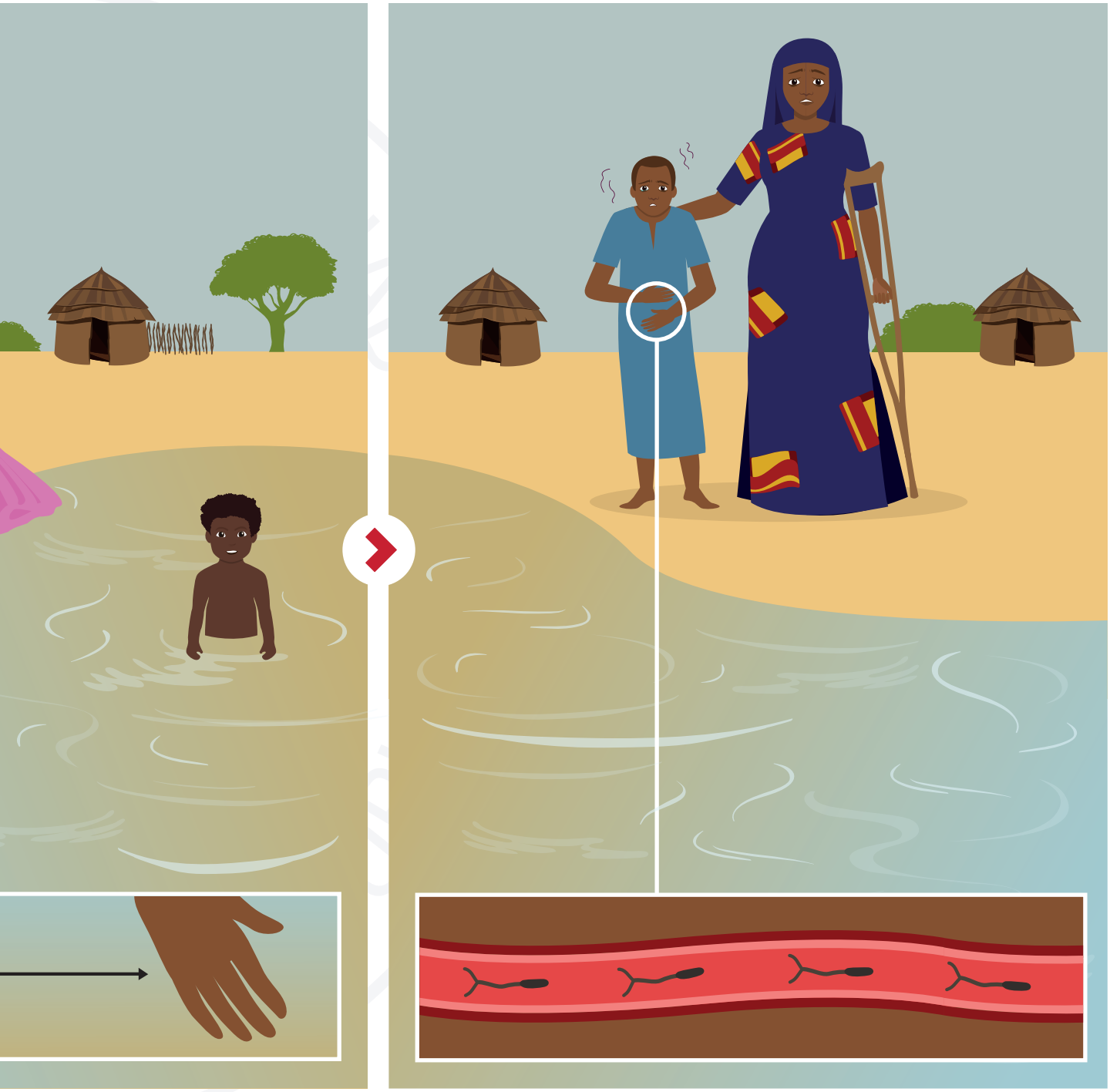

7. Les messages cles a difuser

Vous pouvez utiliser cette page pour avoir les messages importants que vous devez transmettre aux membres de la communauté pendant les causeries que vous allez organiser avant le jour de la distribution des médicaments.

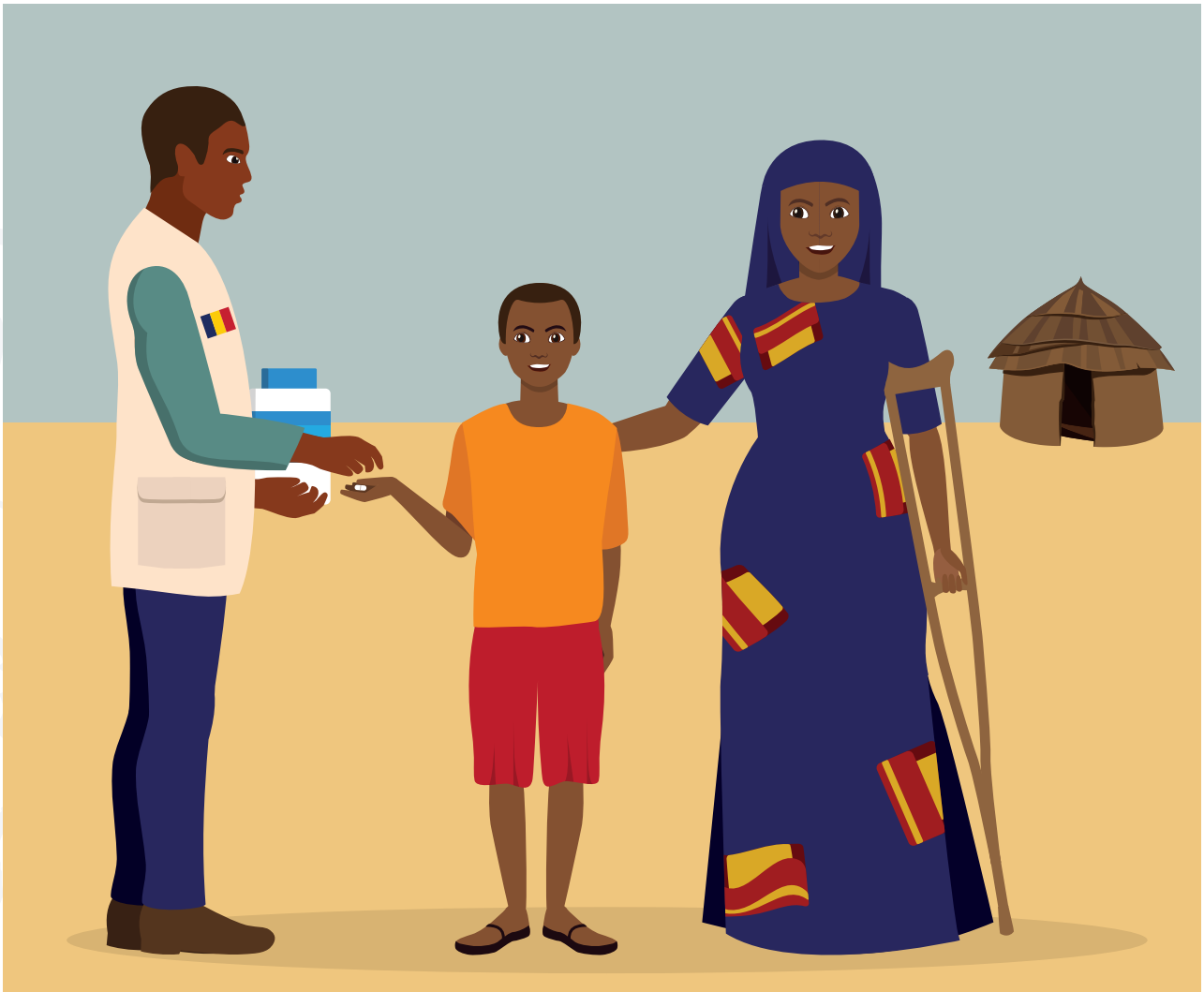

À faire: Accepter de faire prendre les médicaments à vos enfants pendant les campagnes de traitement de masse

---

À éviter: De refuser de faire prendre les médicaments à vos enfants pendant les campagnes de traitement de masse

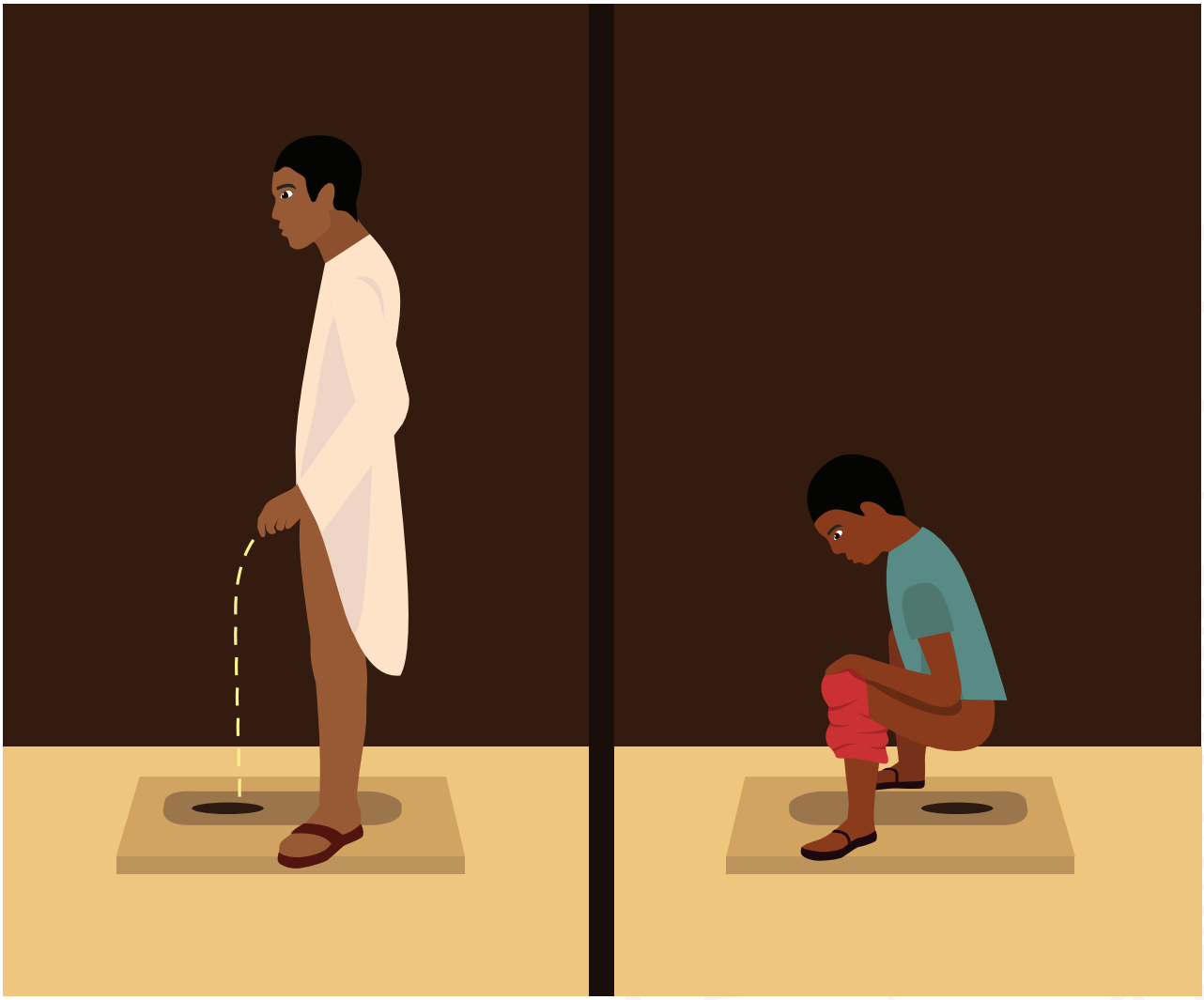

À faire: Utiliser des latrines propres pour aller aux selles ou uriner

À éviter: D’aller aux selles ou d’uriner dans des cours d’eau comme les marigots, les lacs, les fleuves et rivières

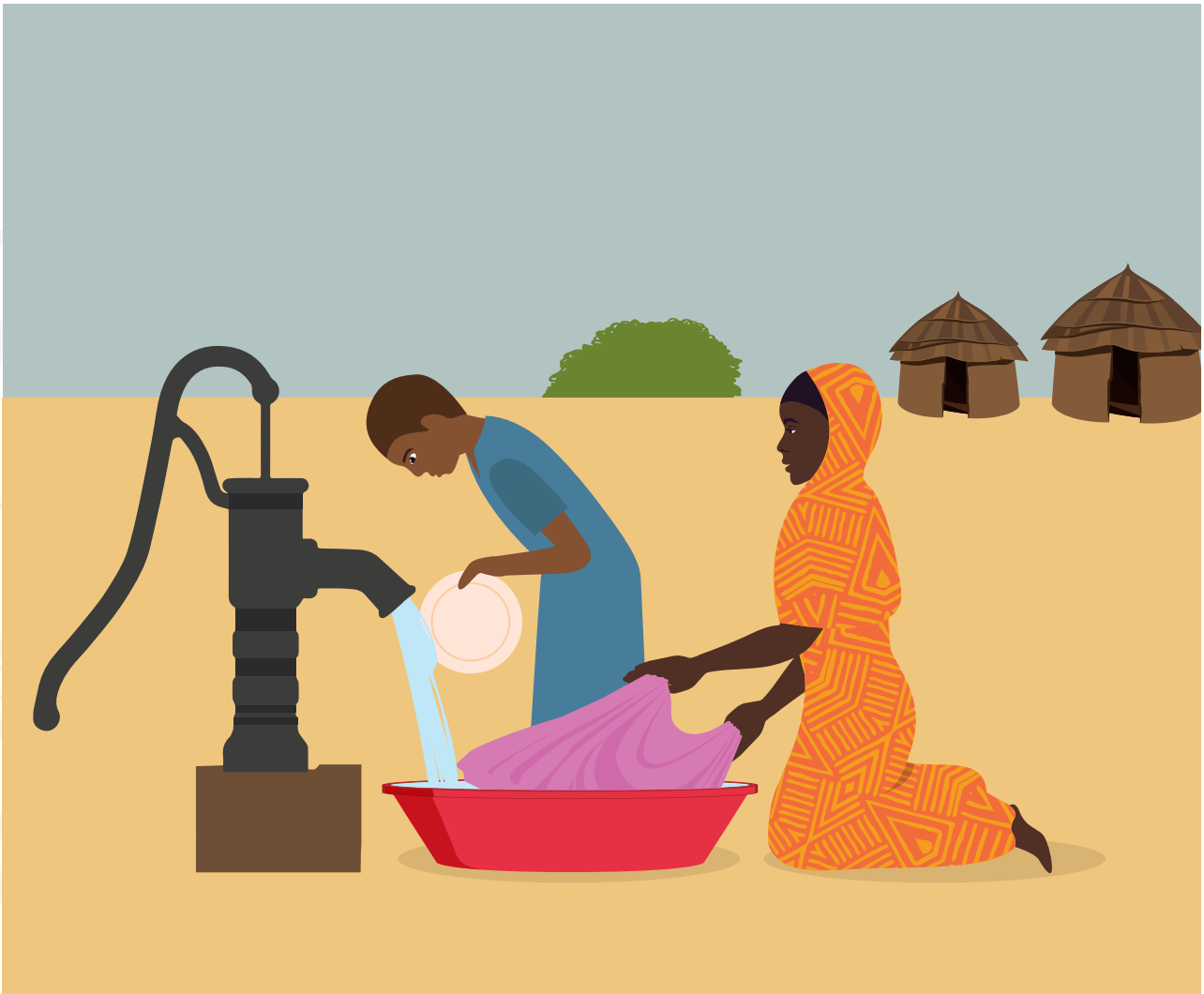

À faire: Faire la lessive ou la vaisselle avec de l'eau potable

---

À éviter: De faire la lessive ou la vaisselle dans un cours d'eau comme les marigots, les lacs, les fleuves et rivières

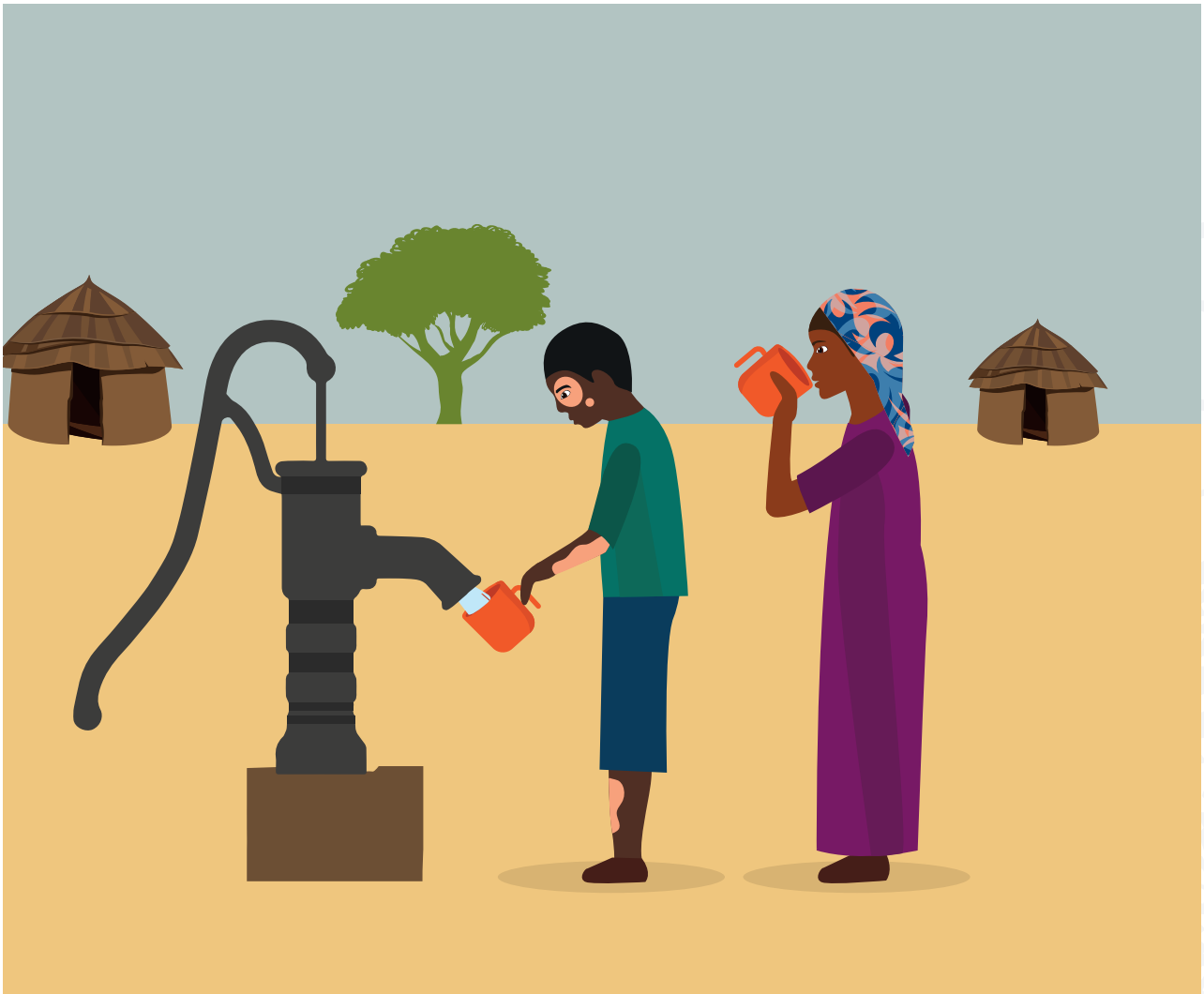

À faire: Toujours boire de l'eau potable

À éviter: De boire l'eau provenant des cours d'eau comme les marigots, les lacs, les fleuves et rivières

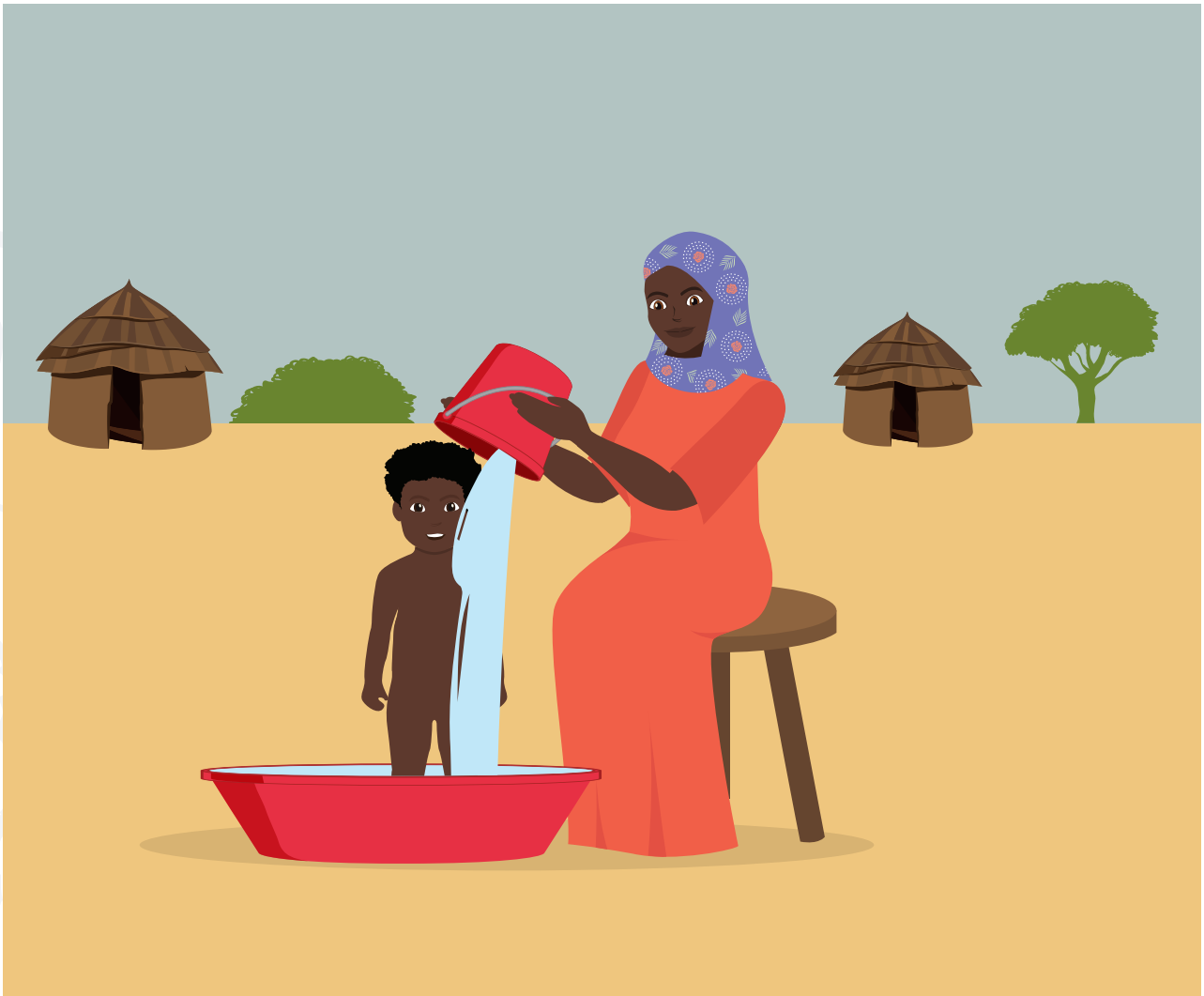

À faire: Interdire a vos enfants de se baigner dans des cours d’eau comme les marigots, les lacs et rivières

---

À éviter: De se baigner dans des cours d’eau comme les marigots, les lacs, les fleuves et rivières

8. Les contacts d’urgence

Incluez une liste de contacts d’urgence, tels que les coordonnées des professionnels de santé locaux ou des services médicaux d’urgence, au cas où des problèmes surviendraient pendant la campagne.

Nom et prénom : ______________________________

Fonction : ____________________________________

Numéro de téléphone : _________________________

Nom et prénom : ______________________________

Fonction : ____________________________________

Numéro de téléphone : _________________________

E DU TCHAD ★ PUBLIQUE
SANTÉ PUBLIQUE

RÉPUBLIQUE DU TCHAD
MINISTÈRE DE LA SANTÉ PUBLIQUE

OPC
EMPOWERING
THROUGH SIGHT

Sightsavers
